# Recurrence patterns and evolution of submicroscopic and asymptomatic *Plasmodium vivax* infections in malaria-endemic areas of the Peruvian Amazon

**DOI:** 10.1371/journal.pntd.0012566

**Published:** 2024-10-31

**Authors:** Stefano S. Garcia Castillo, Caroline Abanto Alvarez, Ángel Rosas-Aguirre, Carlos Acosta, Rodrigo M. Corder, Joaquín Gómez, Mitchel Guzmán, Niko Speybroeck, Alejandro Llanos-Cuentas, Marcia C. Castro, Anna Rosanas-Urgell, Marcelo U. Ferreira, Joseph M. Vinetz, Dionicia Gamboa, Katherine Torres

**Affiliations:** 1 Laboratorio de Malaria: Parásitos y Vectores, Laboratorios de Investigación y Desarrollo, Facultad de Ciencias y Filosofía, Universidad Peruana Cayetano Heredia, Lima, Peru; 2 Research Institute of Health and Society (IRSS), Université Catholique de Louvain, Brussels, Belgium; 3 Instituto de Medicina Tropical Alexander von Humboldt, Universidad Peruana Cayetano Heredia, Lima, Peru; 4 Division of Epidemiology and Biostatistics, University of California, Berkeley School of Public Health, Berkeley, California, United States of America; 5 Department of Parasitology, Instituto of Biomedical Sciences, University of São Paulo, São Paulo, Brazil; 6 Laboratorio ICEMR-Amazonia, Laboratorios de Investigación y Desarrollo, Facultad de Ciencias y Filosofía, Universidad Peruana Cayetano Heredia, Lima, Peru; 7 Harvard T.H. Chan School of Public Health, Harvard University, Boston, United States of America; 8 Institute of Tropical Medicine, Antwerp, Belgium; 9 Global Health and Tropical Medicine, Institute of Hygiene and Tropical Medicine, NOVA University of Lisbon, Lisbon, Portugal; 10 Section of Infectious Diseases, Department of Internal Medicine, Yale School of Medicine, New Haven, Connecticut, United States of America; ISGlobal - IGTP, SPAIN

## Abstract

**Background:**

In the Peruvian Amazon, *Plasmodium vivax* malaria transmission is maintained due to the high frequency of recurrences. By understanding the recurrence rates of submicroscopic and asymptomatic cases, we can develop informed strategies to prevent transmission more efficiently and disrupt the silent transmission cycle.

**Methods:**

A three-year, population-based cohort study was conducted in two sites, Cahuide and Lupuna, within the Loreto region in Peru from 2013 to 2015. The study included 385 individuals and aimed to examine the temporal dynamics of malaria recurrences and their impact on transmission and control.

**Results:**

Individuals from Lupuna presented a higher risk of *P*. *vivax* infections compared to Cahuide, where most recurrences were asymptomatic and submicroscopic. It is estimated that a great proportion of these recurrences were due to relapses in both communities. The application of molecular diagnostic method proved to be significantly more effective, detecting 2.3 times more episodes during the follow-up (PCR, 1068; microscopy, 467). PCR identified recurrences significantly earlier, at 151 days after an initial infection, compared to microscopy, which detected them on average after 365 days. Community, occupation and previous malaria infections were factors associated with recurrences. Finally, potential infection evolution scenarios were described where one frequent scenario involved the transition from symptomatic to asymptomatic infections with a mean evolution time of 240 days.

**Conclusions:**

This study explores the contrast in malaria recurrence risk among individuals from two endemic settings, a consequence of prolonged exposure to the parasite. Through the analysis of the evolution scenarios of *P*. *vivax* recurrences, it is possible to have a more complete vision of how the transmission pattern changes over time and is conditioned by different factors.

## Author summary

The emergence of asymptomatic and lower parasitemia cases has reduced the detection of *P*. *vivax* with conventional surveillance methods. This situation raises questions about whether people without symptoms or with low levels of the parasite can still spread the disease and act as sources of infection. In regions with low transmission, like the Peruvian Amazon, our three-year cohort study can help understand how the pattern of transmission changes over time due to various factors. These factors include the time of recurrences detection, factors associated with recurrence development, and evolution and resolution of infections over time. The analysis of the sociodemographic factors has an important contribution to the understanding of the heterogeneous transmission of malaria in the Amazon. By studying malaria endemic communities over an extended period, we have uncovered a high proportion of people experiencing repeated recurrences of the infection, often without showing any symptoms. We have also confirmed that molecular diagnosis methods are more effective. Additionally, our results emphasize the significance of factors such as community, occupation, and previous malaria infection in recurrence cases. Finally, our findings provide valuable insights into the evolution of infections over time and how external factors influence transmission in endemic areas.

## Introduction

Malaria elimination remains a global public health priority as it affects mainly poor and developing countries. The many unknown aspects of its epidemiology in high heterogenic and low-transmission settings continue to challenge the eradication goal. *Plasmodium falciparum* (*P*. *falciparum*) is responsible for over 90% of malaria deaths, while *Plasmodium vivax* (*P*. *vivax*) is the most prevalent and widespread reaching countries in Asia-Pacific, the Horn of Africa, and across Central and South America [1,2]. Even though there have been enormous efforts to reduce the burden of malaria, *P*. *vivax* is prevalent in countries that are moving towards elimination, as well as in most regions where both *P*. *vivax* and *P*. *falciparum* coexist [2,3]. In 2021, it is estimated that 4.9 million cases occurred by *P*. *vivax*; however, this number might be higher due to underreporting [4]. Many regions have developed intervention methods that are adapted to its context to mitigate the disease; nevertheless, these activities are based on *P*. *falciparum* epidemiology and have less impact on *P*. *vivax* control [2].

*P*. *vivax* has been considered a benign disease, but unlike other species, has certain characteristics that make its control and elimination challenging. First, the presence of a latency phase can cause relapse episodes despite treatment [5]. This dormant stage can produce multiple clinical recurrences from a single infected bite [5]. In high transmission settings, children can rapidly develop naturally acquired immunity against the blood stages [6]. In addition, within the life cycle, there is early and continuous development of gametocytes, appearing before clinical symptoms so that transmission may occur before diagnosis [7]. As a result, the presence of gametocytes from the early stages of infection makes *P*. *vivax* highly transmissible to mosquitoes [1, 5]. Unlike other *Plasmodium* species, the extrinsic incubation period for *P*. *vivax* in the vector is shorter, leading to the early development of infectious sporozoites [8]. Finally, the emergence of asymptomatic and lower parasitemia cases has reduced the detection of *P*. *vivax* with conventional surveillance methods [2]. These features open multiple questions about the infectiousness of asymptomatic and submicroscopic *Plasmodium vivax*-infected individuals and their potential to act as parasitic reservoirs.

For instance, in Peru, intermittent intervention programs, limited access to the health system, the predominance of submicroscopic and asymptomatic infections, and frequent malaria recurrences have hampered the country’s effort to control the disease [9,10]. Previous studies focused on the prevalence of asymptomatic and submicroscopic infections underscore the urgent need for the development and implementation of highly sensitive diagnostic tools that can be easily used in the field [11,12]. Passive case detection uses routine diagnostic methods, such as microscopy and RDTs, which are no longer sensitive enough to detect such low parasitemia levels (< 100 parasites/μL) or patients who do not experience clinical symptoms [12–14]. Therefore, the use of tests such as polymerase chain reaction (PCR), which detects and characterizes parasitemia more accurately, are necessary for blocking residual transmission [14]. These conditions promote individuals to become potential reservoirs of the parasite [15,16].

Malaria incidence in Peru has recently decreased, and the current annual incidence of asymptomatic infections represents 73% of detected cases, with submicroscopic infections ranging between 68% to 74% [16–18]. In addition, many studies in the Amazonia have reported alarming recurrences rates, defined as the reappearance of asexual parasites after treatment [19–21]. *Plasmodium vivax*, unlike other species, has a latent hepatic phase in the form of hypnozoites that can produce a relapse month or even years after the primary infection. The characteristics of recurrences, especially the ones only detectable by molecular methods, during long rigorous follow-up periods still need to be fully understood. The ICEMR Amazonia cohort collected valuable and relevant information during three years of monthly follow-up of individuals from two different settings in the Peruvian Amazon: Lupuna and Cahuide, that will allow to investigate the complexity of the disease’s transmission in this regional context [22–24]. This study estimates the time of detection of recurrences, their average duration, and other characteristics such as evolution and associated factors that will aid in controlling residual malaria in the Peruvian Amazon region.

## Methods

### Ethics

The study "Impact of asymptomatic carriers on the epidemiology and control of malaria in the Peruvian Amazon" was reviewed and approved by the Ethical Committee of the Universidad Peruana Cayetano Heredia, Lima, Peru (SIDISI code: 57395). All participants provided written informed consent for participation in the study, including future use of their samples in studies of antimalarial antibody responses. For child participants, written parental consent and the child’s assent were obtained. Participants who did not provide informed consent for blood sample use were excluded from the study.

### Study area

**Fig 1:**
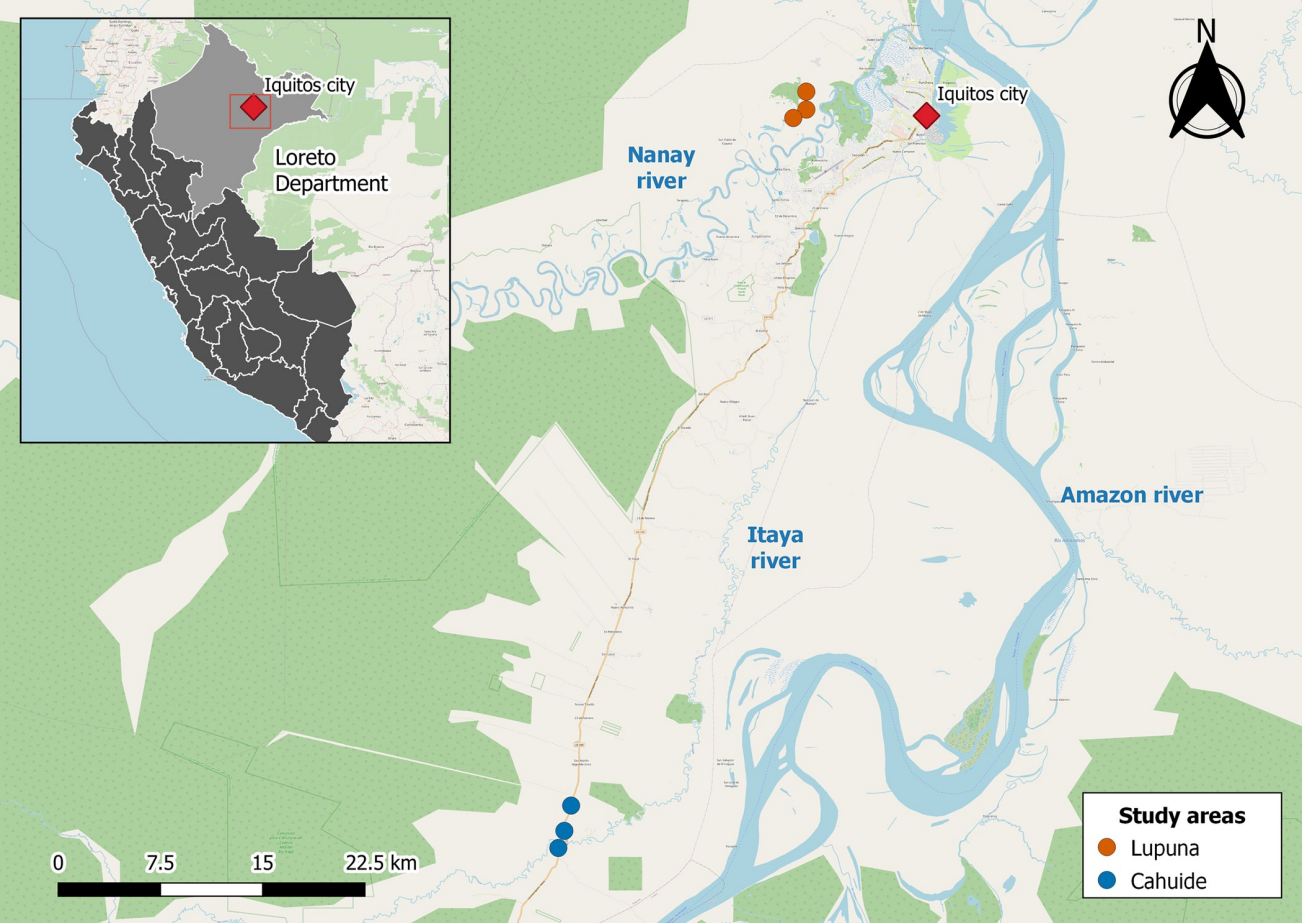
Map of Study Areas. The collection sites in the Lupuna area (Santa Rita, San José de Lupuna, and San Pedro) are denoted by orange circles. Likewise, collection sites in the Cahuide area (Habana, Doce de Abril, and Cahuide village) are denoted by blue circles. The city of Iquitos and the rivers commonly used for transportation are highlighted in red and blue, respectively. The map was generated using QGIS 3.16 (QGIS Development Team, 2023. QGIS Geographic Information System. Open-Source Geospatial Foundation Project. http://www.qgis.org/) and utilizes layers from OpenStreetMap (http://www.openstreetmap.org/), which is licensed under the Open Data Commons Open Database License (ODbL). Shapefiles are freely available on the Peruvian National Institute of Statistics and Informatics (INEI) website (https://ide.inei.gob.pe/).

The study was conducted in two communities, Cahuide (CAH) and Lupuna (LUP), in the northeastern Loreto region of Peru (Fig 1). Cahuide (04°13–785’S, 73°276’W) is located on both sides of the Iquitos-Nauta highway. Lupuna (03°44–591’S, 73°19–615’W) is in the Iquitos district, and its only access is through the Nanay River. From December to May is typically marked by tropical rainfall, whereas from June to November is generally associated with dry conditions in the area [25].

### Study design

This prospective longitudinal cohort study was conducted by the Amazonia International Center of Excellence in Malaria Research (Amazonia ICEMR) project from January 2013 to December 2015 in two communities in the Peruvian Amazon. After the census in July and August 2012, the baseline parasitological survey was conducted in September and October 2012, followed by enrollment in November and December 2012 [22]. Residents from Cahuide and Lupuna communities older than 3 years of age were invited to participate after signing informed consent or assent. Monthly follow-ups for the entire cohort were performed by active case detection (ACD) and passive case detection (PCD). All patients completed a survey on clinical and socio-demographic data. In addition, a blood sample was collected by digital puncture on a slide for microscopic diagnosis and on filter paper (Whatman grade 3, 340 Whatman, Springfield Mill, USA) for later molecular diagnosis by quantitative real-time polymerase chain reaction (PCR). During the ACD, individuals with *Plasmodium* infection detected by microscopy were referred to the nearest health post for treatment; while, during PCD, individuals were treated at the health post immediately. In both cases, active weekly follow-up was performed for one month. The treatment schedule for all symptomatic cases of malaria and microscopy positives, according to Ministry of Health guidelines, was chloroquine for three days (10 mg/g for days 1 and 2, and 5 mg/kg for day 3) and primaquine for 7 days (0.5 mg/kg/day) for *P*. *vivax* infections.

Microscopy diagnosis (MIC) was performed by thick and thin blood smears, where the slides were stained with 10% Giemsa solution. One slide was promptly examined on-site, while the second one was read the following day at the reference laboratory in Iquitos to confirm the diagnosis. It was considered negative if no presence of the parasite was found after examining 100 microscopic fields. For molecular diagnosis, DNA was extracted from the blood samples impregnated in filter paper (~6 mm^2^-pieces) using a commercial kit (QIAamp DNA Blood Minikit, Qiagen, Hilden, Germany). Molecular diagnosis was based on the detection of the *Plasmodium* 18s rRNA gene by polymerase chain reaction (PCR) described by Mangold et al. [26]. A comprehensive review of the study’s methodology, as well as the prevalence, incidence and transmission dynamics of malaria during the baseline assessment and cohort phase, has been conducted by Rosas-Aguirre et al. in 2017 [22] and 2020 [23].

This study included 385 individuals who met all the following criteria: i) they were diagnosed with microscopic *P*. *vivax* infection for the first time during the cohort period, which was considered as "*P*. *vivax* initial infection" from now on, ii) they received immediate treatment after the *P*. *vivax* initial infection, iii) they did not have a new positive diagnosis (PCR and microscopy) for *P*. *vivax* during the following 21 to 45 days, indicating treatment effectiveness, iv) they had monthly microscopy and PCR diagnostics available from the time of the *P*. *vivax* initial infection to the end of the cohort study.

### Definitions

For this study, a microscopic infection is defined as one in which, in addition to being PCR +, parasites are detected by MIC. Submicroscopic infection is defined as one in which no parasites were detected by MIC but was PCR +. On the other hand, the clinical status of a *P*. *vivax* infection is asymptomatic when there is no fever, headache and chills (malaria triad); or symptomatic if at least one of these symptoms is present. Furthermore, the initial *P*. *vivax* infection of an individual detected is defined as the first intervention in which the individual had a positive diagnosis for *P*. *vivax* by PCR and microscopy; in addition, the initial infection is independent of the clinical status of the infection as it can be symptomatic or asymptomatic. Finally, a *P*. *vivax* recurrence is defined as an episode where the parasite is detected in blood after previous positive diagnosis, treatment and at least one negative diagnosis after treatment. Recurrences can be recrudescence (treatment failure), reinfection, or relapses. Also, recurrences may have a different clinical status (asymptomatic or symptomatic) and can be detected by microscopy or not (microscopic or submicroscopic).

### Statistical analysis

Statistical analysis of the data was performed using the statistical program R (version 4.0.0) on the RStudio platform, using the packages: “rstatic”, “stats”, “survival”, and “survminer”. Comparisons of sociodemographic and epidemiological characteristics between communities were carried out with Fisher’s exact test. Survival analysis (Kaplan-Meier) was performed to determine the probability of remaining free of recurrence. The following outcomes were considered: time to recurrence detected by microscopy, and time to recurrence detected by PCR. The probability of having an asymptomatic and symptomatic recurrent event over time was assessed by Nelson-Allen risk analysis. In addition, this analysis was also performed to find the probability of risk of recurrence by community of residence, sex, age group, occupation and number of previous malaria episodes. Raw data for all figures are included in S1 Data.

The Cox Counting Process (CP) model was used for unadjusted and multivariable analysis to identify factors associated with recurrence detected by PCR according to characteristics such as community, age group, occupation and number of previous malaria episodes. The analysis of factors associated with asymptomatic/symptomatic and microscopic/submicroscopic recurrences was performed. Variables associated with a p-value < 0.2 were included in the multivariable analysis, which was adjusted for confounding factors. The proportional hazards assumption was checked.

**Fig 2:**
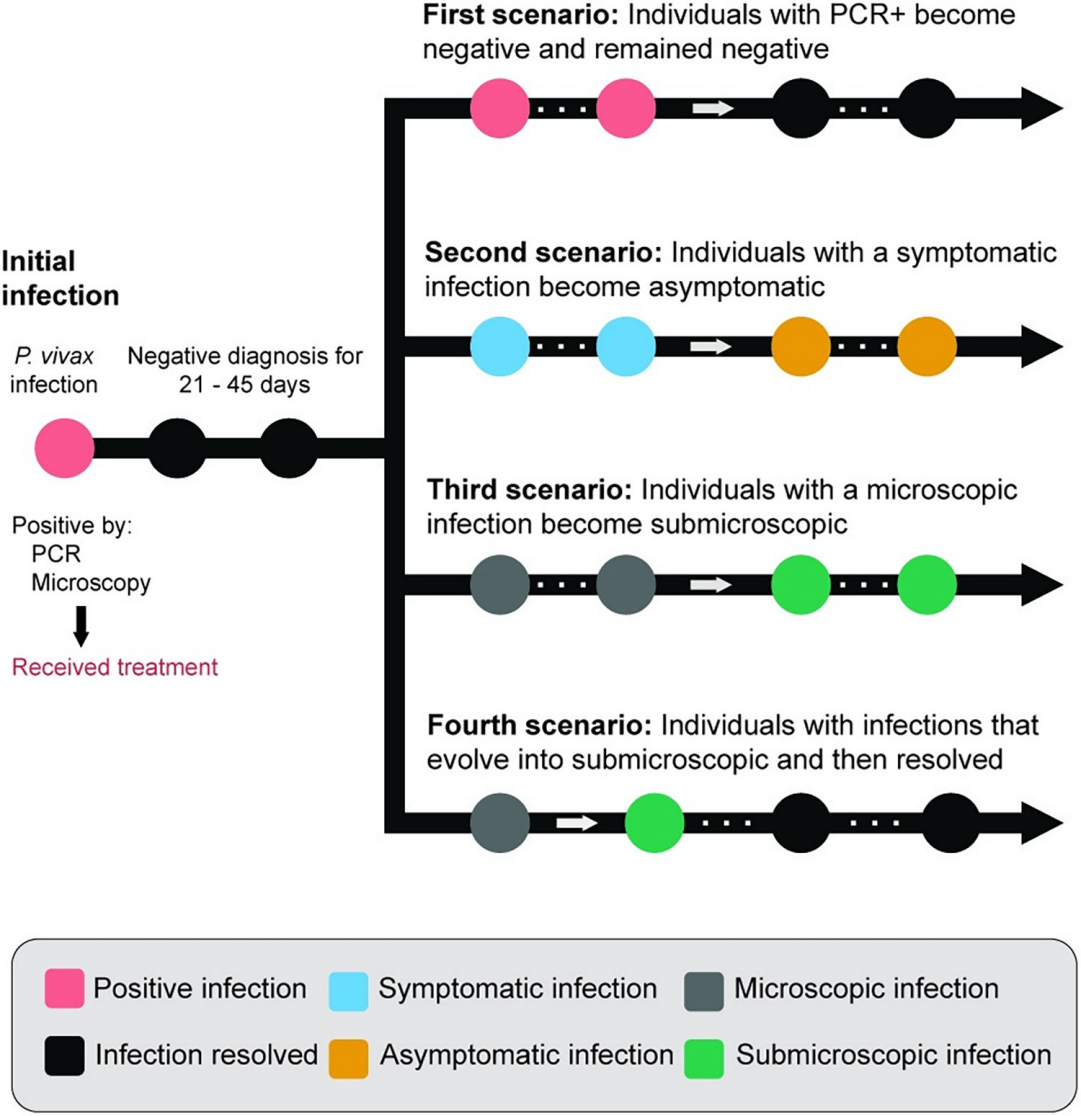
Diagram illustrating the evolution scenarios of *P*. *vivax* through recurrent episodes.

The dynamics of recurrence infections were described in eight scenarios. The frequency of these scenarios was calculated using the incidence rate, determined by estimating the number of new cases and the total number of person-years at risk. The initial four scenarios centered on individuals who experienced a single type of infection consistently during the entire follow-up period: (i) microscopic and symptomatic infections, (ii) microscopic and asymptomatic infections, (iii) individuals without treatment and only asymptomatic infections, and (iv) individuals with mixed microscopic/submicroscopic and asymptomatic/symptomatic infections. The evolution of the recurrent infections of the individuals was classified from four main scenarios during the three years of the study (Fig 2). After the initial *P*. *vivax* infection detected by positive PCR and microscopy, these scenarios are described: (i) an individual with recurrent PCR+ episodes evolves to resolve the infection and remain negative, (ii) an individual with recurrent symptomatic infections evolves to have only asymptomatic infections, (iii) an individual with recurrent microscopic infections evolves to have only submicroscopic infections and, (iv) individual who started to develop submicroscopic infections evolves to resolves and remain negative. These patterns of change were identified by outlier analysis (quantile (0.75) + IQR) (S1–S4 Figs). Survival analysis was also performed to determine the time elapsed for the evolution or resolution of infection for each scenario.

### Parametric model

Here we use a simple modeling approach to capture the dynamics of *P*. *vivax* recurrences and to estimate the proportion of them that are due to relapses and new infections. Under the assumption that hypnozoites have a constant activation rate over time, relapses have previously been parametrically modelled by exponential survival functions [27]. Here we use a similar framework and assume that hypnozoites have a constant activation rate *λ*. As only patients with hypnozoites may experience this event, we also assume that only a fraction *p* of the population is susceptible to relapses. Notice that the proportion *p* is not expected to vary between study sites. We also assume a constant infection rate *β* to describe the dynamics of new infections. Different than *p* and *λ*, the infection rate *β* is expected to be site-specific. Due to the competing risk framework, the fraction *p* of the population that is susceptible to relapses comprises those who actually relapse and those who would eventually relapse but instead have a new infection (competing event) first. Assuming that all enrolled patients are susceptible to new infections and that recurrences started to be detected 50 days after the index vivax episode, the site-specific parametric survival functions *S*_*C*_(*t*;*p*,*λ*,*β*_*C*_) and *S*_*L*_(*t*;*p*,*λ*,*β*_*L*_) can be written as,

$$\left\{ \begin{array}{l} S_{C}(t;p,\lambda ,\beta_{C})=(1-p)e^{-(\beta_{C})(t-50)}+pe^{-(\lambda +\beta_{C})(t-50)}\\ S_{L}(t;p,\lambda ,\beta_{L})=(1-p)e^{-(\beta_{L})(t-50)}+pe^{-(\lambda +\beta_{L})(t-50)} \end{array}\right.$$ (1)

where subindices *C* and *L* represent values associated with Cahuide and Lupuna, respectively.

To fit each competing risk survival model from Eq 1 to empirical data, we consider the following generic survival analysis framework. Let *Y* be the time to malaria recurrence (either relapse or new infection) with survival distribution function S(∙,***θ***) and associated density function *f*(∙,***θ***), where ***θ*** is a parameter vector (i.e., ***θ*** = (*p*,*λ*,*β*)). Let *Z* be a censoring event (in our case, a *P*. *falciparum* infection or the end of follow-up) such that the observable time variable (*X*) is the minimum between *Y* and *Z* (that is, *X* = *Z* in case of a censoring event and *X* = *Y* in case of a malaria recurrence); and let δ be an indicator variable denoting whether a time of malaria episode or a time of censoring was observed. The actual data to be observed for each subject may then be mathematically represented by,

X=min(Y,Z)andδ=I(X=Y). (2)

Assuming independent and non-informative censoring, the likelihood function for the underlying survival model for inference on ***θ***, based on all *N* individuals, is defined as follows,

$$l(\theta |(X,\delta ))=\prod_{i=1}^{N}{\left[f(X_{i},\theta )\right]}^{\delta_{i}}∙{\left[S(X_{i},\theta )\right]}^{1-\delta_{i}},$$ (3)

with ***X*** = (*X*_1_,…,*X*_*n*_) being the vector of all *N* individuals observed time variables and ***δ*** = (*δ*_1_,…,*δ*_*n*_), the vector of all *N* individuals indicator variables (each of them as defined by Eq 2). Because *p* and *λ* are assumed to be invariant across study sites and the infection rates *β*_*C*_,*β*_*L*_ are site-specific, we maximized the product *l*_*C*_∙*l*_*L*_ of the likelihoods that best explain Cahuide and Lupuna observed data (with the likelihood *l* as presented in Eq 3), respectively, over parameters *p*,*λ*,*β*_*C*_,*β*_*L*_. To obtain the maximum likelihood estimates and 95% credible intervals for each parameter, we used Markov Chain Monte Carlo (MCMC) methods considering 10^5^ iterations. The parameter estimations and respective 95% CI were obtained from the MCMC posterior distributions.

## Results

### Socio-demographic characteristics of the study population

A total of 385 individuals (CAH, 118; LUP, 267) distributed in 207 households (CAH, 74; LUP, 133) were included in this study. The proportions of sex, age group, educational level, and outdoor occupation did not differ between communities (p-value>0.05). Individuals who had more than 4 episodes of malaria in their lifetime was higher in LUP (22.5%) than in CAH (13.6%) (p-value = 0.023); however, experiencing malaria in the previous year of the study was more common in CAH than in LUP (p-value<0.0001) (Table 1). Likewise, between communities, the difference in parasite exposure was also influenced by the characteristics of their houses (Table 2). Between the two communities, CAH had a higher percentage of households with better resources and infrastructure, such as availability of drinking water (CAH, 36.5%; LUP, 14.6%; p<0.0001), electricity (CAH, 63.5%; LUP, 25.6%; p<0.0001), wooden floors (CAH, 45.9%; LUP, 11.3%; p<0.0001) and less use of the field as a toilet (CAH, 25.7%; LUP, 43.6%; p = 0.03). However, Lupuna had a higher number of bed nets (CAH, 55.1%; LUP, 88.7%; p<0.0001), but for both communities very few nets were insecticide-treated net.

**Table 1:** Socio-demographic and epidemiological characteristics of study participants in the communities of Cahuide and Lupuna according to baseline survey.

	Total		Cahuide		Lupuna		*p-value*
	n	%	n	%	n	%	
**Sex**							
Female	215	55.8	65	55.1	150	56.2	0.91
Male	170	44.2	53	44.9	117	43.8	
**Age group**							
≤ 15 years	179	46.5	59	50.0	120	44.9	0.38
> 15 years	206	53.5	59	50.0	147	55.1	
**Education**							
None or preschool	47	12.2	17	14.4	30	11.2	0.20
Elementary school	210	54.5	70	59.3	140	52.4	
High school	125	32.5	30	25.4	95	35.6	
University or higher education	3	0.8	1	0.8	2	0.7	
**Outdoor occupation (logger, fisherman o farmer)**							
Yes	73	19.0	16	13.6	57	21.3	0.09
No	312	81.0	102	86.4	210	78.7	
**Time living in the community**							
< 2 years	40	10.4	24	20.3	16	6.0	**<0.0001**
2–10 years	140	36.4	48	40.7	92	34.5	
> 10 years	205	53.2	46	39.0	159	59.6	
**Travel in the last month (more than 10 km. from the community)**							
Yes	3	0.8	2	1.7	1	0.4	**0.22**
No	382	99.2	116	98.3	266	99.6	
Malaria episodes by *Plasmodium vivax* in your lifetime *							
0	132	34.4	42	35.6	90	33.8	**0.023**
1	79	20.6	34	28.8	45	16.9	
2–3	97	25.3	26	22.0	71	26.7	
≥ 4	76	19.8	16	13.6	60	22.6	
Malaria episodes by *Plasmodium vivax* in the last year **							
0	285	74.4	58	49.2	227	85.7	**<0.0001**
1	72	18.8	44	37.3	28	10.6	
2–3	23	6.0	13	11.0	10	3.8	
≥ 4	3	0.8	3	2.5	0	0	

One individual with missing data (Total N = 384, Lupuna N = 266). Two individuals with missing data (Total N = 383, Lupuna N = 265). (*) One individual with missing data (Total N = 348, Lupuna N = 266). (**) Two individuals with missing data (Total N = 383, Lupuna N = 265).

**Table 2:** Basic characteristics of study participants’ households according to baseline survey.

	Total		Cahuide		Lupuna		*p-value*
	n	%	N	%	n	%	
**Overcrowding (>3 people/bedroom)**							
Yes	36	17.4	15	20.3	21	15.8	0.45
No	171	82.6	59	79.7	112	84.2	
**Wall material**							
Mat, Palm	12	5.8	5	6.8	7	5.3	**0.06**
Plastic	6	2.9	3	4.1	3	2.3	
Wood	174	84.1	65	87.8	109	82.0	
Brick	15	7.2	1	1.4	14	10.5	
**Floor material**							
Soil	116	56.0	29	39.2	87	65.4	**<0.0001**
Cement	41	19.8	10	13.5	31	23.3	
Wood	49	23.7	34	45.9	15	11.3	
Others	1	0.5	1	1.4	0	0	
**Roof material**							
Palm	155	74.9	63	85.1	92	69.2	0.01
Tin, Plastic	52	25.1	11	14.9	41	30.8	
**Electricity**							
Yes	81	39.1	47	63.5	34	25.6	**<0.0001**
No	126	60.9	27	36.5	99	74.4	
**Potable water for drinking**							
Yes	46	22.2	27	36.5	19	14.3	**<0.0001**
No	161	77.8	47	63.5	114	85.7	
**Water source**							
Public water system	4	1.9	3	4.1	1	0.8	**0.01**
Communal water faucet	63	30.4	21	28.4	42	31.6	
Rivers	58	28.0	21	28.4	37	27.8	
Well	77	37.2	24	32.4	53	39.8	
Missing data	5	2.4	5	6.8	0	0	
**Sanitary Installation**							
Pit latrine	48	23.2	21	28.4	27	20.3	**0.03**
Ground hole, cesspool	82	39.6	34	45.9	48	36.1	
Field, rivers, others	77	37.2	19	25.7	58	43.6	
**Garbage disposal**							
Burning	69	33.3	25	33.8	44	33.1	**0.18**
Buring	26	12.6	14	18.9	12	9,0	
Field, river	107	51.7	34	45.9	73	54.9	
Missing data	5	2.4	1	1.4	4	3.0	
**Fuel for cooking**							
Gas	7	3.4	1	1.4	6	4.5	**<0.0001**
Kerosene, charcoal	15	7.2	13	17.6	2	1.5	
Firewood	184	88.9	60	81.1	124	93.2	
Missing data	1	0.5	0	0.0	1	0.8	
**Coverage mosquitos net (mosquitos’ nets/beds), %.**							
<80	8	4.6	3	4.1	5	3.8	1.00
≥80	199	96.1	71	95.9	128	96.2	
**Bed net material**							
Coarse cotton	36	17.4	29	39.2	7	5.3	**<0.0001**
Long-lasting insecticide-treated net	13	6.3	5	6.8	8	6.0	
Insecticide-untreated net	156	75.4	38	51.4	118	88.7	
Others	2	1.0	2	2.7	0	0.0	

### Characteristics of recurrent *Plasmodium vivax* infections

Microscopy detection showed that 40.7% and 60.3% of individuals in CAH and LUP, respectively, had at least one recurrence, whereas, with PCR diagnosis, these proportions increased significantly to 76.3% and 83.5% for CAH and LUP. PCR revealed three times more individuals with four or more recurrences than microscopy (125 by PCR and 37 by MIC), with most of these recurrences occurring 180 days after the primary *P*. *vivax* infection (Table 3). Based on the clinical status of recurrence detected by PCR, we found that 82.7% (259/285) of individuals developed asymptomatic recurrences. A great proportion of individuals (22.8%, 59/259) had over four asymptomatic recurrences during the follow up compared to symptomatic recurrences (8.3%, 18/218), indicating that a significant number of individuals are experiencing recurrent infections without exhibiting any symptoms. Moreover, 75.4% (236/313) of individuals had at least one submicroscopic recurrence, with 66.1% (397/601) of these recurrences detected 180 days after the primary *P*. *vivax* infection (Table 3).

**Table 3:** Characteristics of *P*. *vivax* recurrences detected between January 2013 and December 2015.

	Cahuide		Lupuna		Total	
	N	%	N	%	N	%
Number of individuals	118	100	267	100	385	100
**Recurrences detected by PCR**						
Individuals with recurrences	**90**	**76.3**	**223**	**83.5**	**313**	**81.3**
1 recurrence	41	45.6	48	21.5	89	28.4
2 recurrences	14	15.6	32	14.3	46	14.7
3 recurrences	13	14.4	40	17.9	53	16.9
4+ recurrences	22	24.4	103	46.2	125	39.9
Total number of recurrences	233		835		1068	
Number of recurrences/individuals (Median [IQR]; max.)	1 [1–3], 14		3 [1–4], 14		2 [1–4], 14	
Time to detect recurrences						
0–90 days	42	18.0	91	10.9	133	12.5
90–180 days	47	20.2	128	15.3	175	16.4
>180 days	144	61.8	616	73.8	760	71.2
**Recurrences detected by microscopy**						
Individuals with recurrences	**48**	**40.7**	**161**	**60.3**	**209**	**54.3**
1 recurrence	31	64.6	60	37.3	91	43.5
2 recurrences	8	16.7	38	23.6	46	22.0
3 recurrences	6	12.5	29	18.0	35	16.7
4+ recurrences	3	6.3	34	21.1	37	17.7
Total number of recurrences	82		385		467	
Number of recurrences/individuals (Median [IQR]; max.)	nnn[0–1], 8		1 [0–2], 7		1 [0–2], 8	
Time to detect recurrences						
0–90 days	26	31.7	60	15.6	86	18.4
90–180 days	15	18.3	61	15.8	76	16.3
>180 days	41	50.0	264	68.6	305	65.3
**Number of individuals**	**90**	**100**	**223**	**100**	**313**	**100**
**Asymptomatic recurrences**						
Individuals with recurrences	**76**	84.4	**183**	82.1	**259**	82.7
1 recurrence	37	48.7	67	36.6	104	40.2
2 recurrences	20	26.3	45	24.6	65	25.1
3 recurrences	6	7.9	25	13.7	31	12.0
4+ recurrences	13	17.1	46	25.1	59	22.8
Total number of recurrences detected	162		489		651	
Number of recurrences/individuals (Median [IQR]; max.)	2 [1–2.25], 9		2 [1–3.5], 12		2 [1–3], 12	
Time to detect recurrences						
0–90 days	26	16.0	44	9.0	70	10.8
90–180 days	33	20.4	68	13.9	101	15.5
>180 days	103	63.6	377	77.1	480	73.7
**Symptomatic recurrences**						
Individuals with recurrences	**45**	50.0	**173**	77.6	**218**	69.6
1 recurrence	34	75.6	80	46.2	114	52.3
2 recurrences	6	13.3	46	26.6	52	23.9
3 recurrences	4	8.9	30	17.3	34	15.6
4+ recurrences	1	2.2	17	9.8	18	8.3
Time to detect recurrences	71		346		417	
Number of recurrences/individuals (Median [IQR]; max.)	1 [1–1], 13		2 [1–3], 9		2 [1–3], 13	
Time to detect recurrences						
0–90 days	16	22.5	47	13.6	63	15.1
90–180 days	14	19.7	60	17.3	74	17.7
>180 days	41	57.7	239	69.1	280	67.1
**Submicroscopic recurrences**						
Individuals with recurrences	**73**	81.1	**163**	73.1	**236**	75.4
1 recurrence	39	53.4	59	36.2	98	41.5
2 recurrences	15	20.5	32	19.6	47	19.9
3 recurrences	6	8.2	27	16.6	33	14.0
4+ recurrences	13	17.8	45	27.6	58	24.6
Total number of recurrences	151		450		601	
Number of recurrences/individuals (Median [IQR]; max.)	1 [1–3], 9		2 [1–4], 11		2 [1–3], 10	
Time to detect recurrences						
0–90 days	9	6.0	31	6.9	40	6.7
90–180 days	13	8.6	67	14.9	80	13.3
>180 days	45	29.8	352	78.2	397	66.1

Symptoms of individuals with *P*. *vivax* recurrences were recorded (S1 Table and S5 Fig), and headache was in 93.53% of symptomatic recurrences, while 67.39% presented fever. Asymptomatic recurrences were also evaluated for other secondary symptoms such as back pain (1.69%, 11/651), abdominal pain (0.61%, 4/651), cough (1.23%, 8/651) and dizziness (1.08%, 7/651). Submicroscopic recurrences were characterized by headaches, which accounted for the largest percentage (20.47%, 123/601) of symptoms, while fever and chills occurred in 9.15% and 6.82% of cases, respectively.

**Fig 3:**
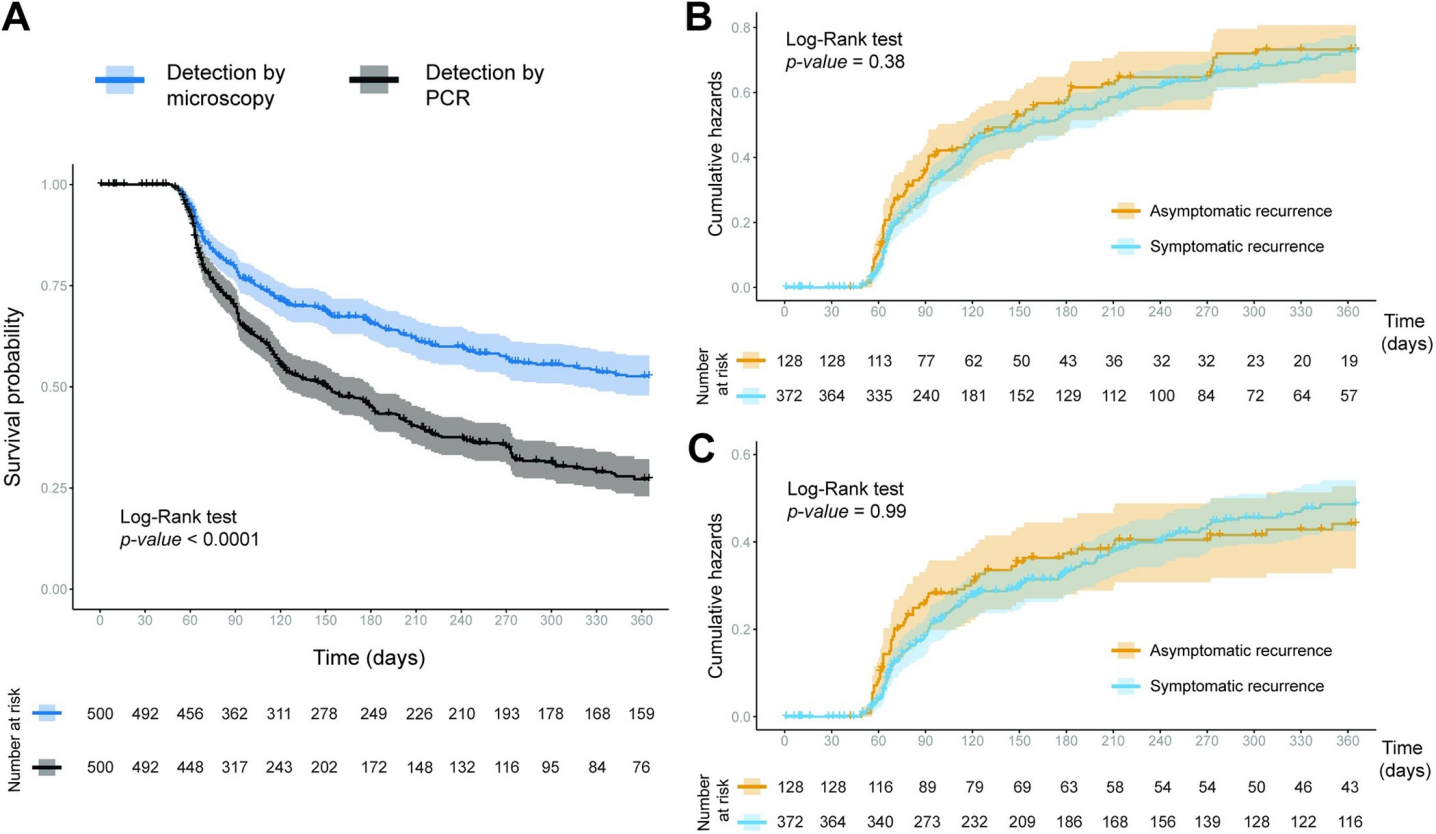
Probability of remaining free of *Plasmodium vivax* recurrence. **(A)** Kaplan-Meier probability of remaining free of the first *P*. *vivax* recurrences according to the detection method. Nelson-Aalen’s risk probability of presenting recurrence of *Plasmodium vivax* detected by PCR **(B)** and microscopy **(C)** according to clinical status (symptomatic, asymptomatic). Comparison between survival and risk curves was analyzed with the Log-Rank test.

The Kaplan–Meier analysis showed that the median time to remain free of recurrence was above 365 days (12 months) by MIC and 151 days (5 months) by PCR (p-value<0.0001, Fig 3). The probability of remaining recurrence-free at day 180 was shown to be 65.7% (95% CI [61.4; 70.3]) when MIC was used for diagnosis, and 44.9% (95% CI [40.5; 49.8]) when PCR was used (Fig 3A). These results suggest half of the cohort experienced their first PCR-detected recurrence by the fifth month of follow-up, compared to 12 months with only MIC diagnosis. Considering the clinical status of PCR-detected recurrences, the probability of having a symptomatic episode by day 180 was 45.8% (95% CI [40.7, 51.6]) and 41.3% (95% CI [33.3, 51.4]) for asymptomatic infections (Fig 3B). Following this trend, microscopic detection revealed no significant differences between the risk curves for symptomatic and asymptomatic recurrences (p-value = 0.99, Fig 3C). Other factors of the individual such as community of origin, presence of outdoor activities and previous malaria episodes in the last year are associated with the occurrence of recurrences (S6 and S7 Figs).

### Factors associated with *P*. *vivax* recurrent infections

**Fig 4:**
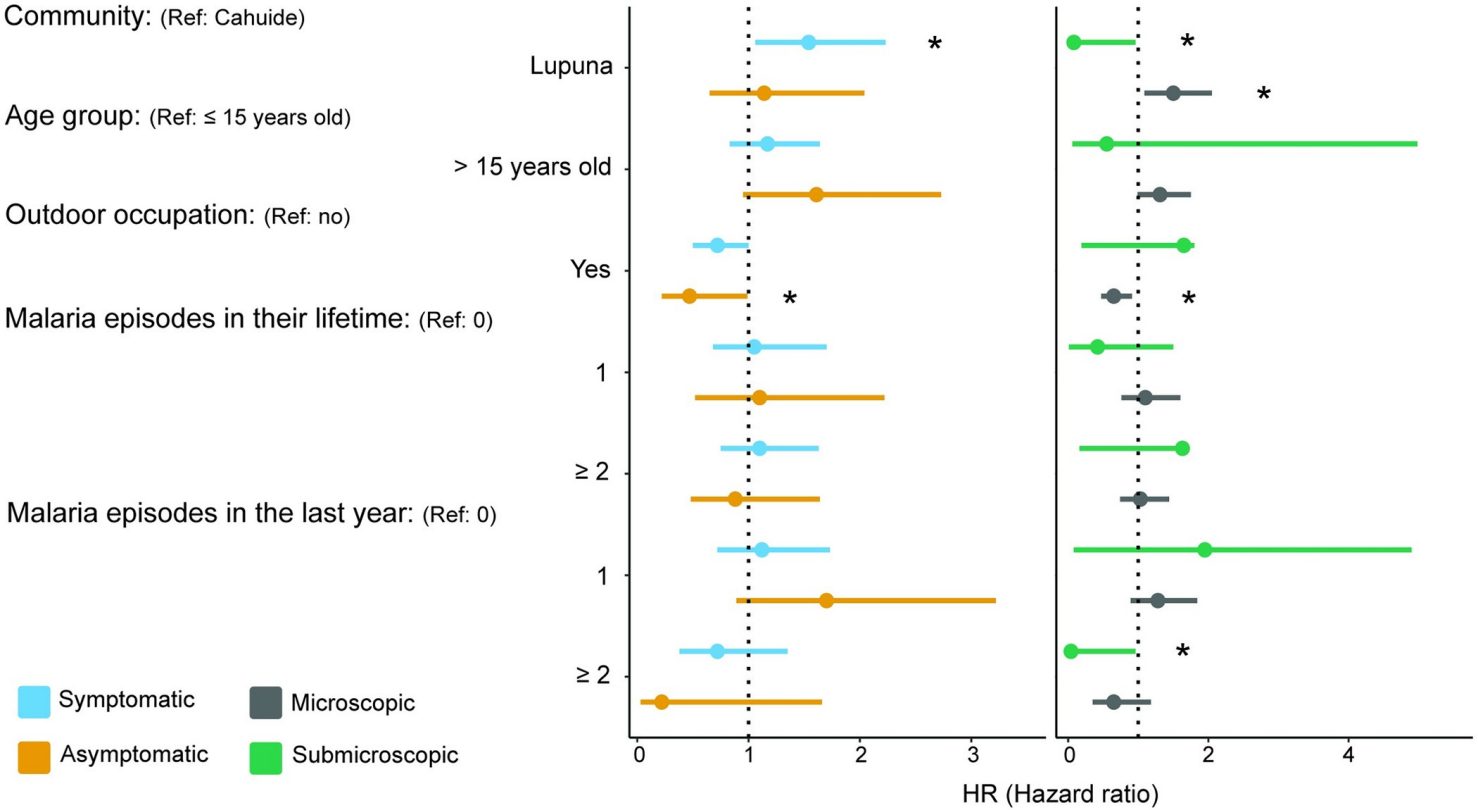
Forest plot of hazard ratios of risk factors associated with *Plasmodium vivax* recurrences. By asymptomatic and symptomatic (yellow and sky-blue points), submicroscopic and microscopic (green and grey points). Significant difference between sites: *, p-value < 0.05.

Factors such as community, sex, age group, outdoor occupation, and previous episodes of *Plasmodium vivax* malaria were considered as potential contributors to recurrent infections. We found that living in LUP is associated with an increased risk of both symptomatic (HR = 1,6 [1.1–2.1], p-value = 0.006) and microscopic (HR = 1.4 [1.1–1.9], p-value = 0.009) recurrences (S2 Table). For submicroscopic recurrences, being older than 15 years old and having had 2 or more malaria episodes in their lifetime were protective factors, reducing the risk by 71% (95% CI [0.091–0.89], p-value = 0.031) and 70% (95% CI [0.091–0.97], p-value = 0.044), respectively (S2 Table). The multivariate analyses included all previously analyzed variables except sex (Fig 4 and S3 Table). Residing in LUP remained as a risk factor for symptomatic and microscopic recurrences, with an increase of 54% (95% CI [1.06; 2.23], p-value = 0.024), and 50.0% (95% CI [1.09; 2.05], p-value = 0.011) respectively. However, this variable showed protective associations against submicroscopic recurrences (HR = 0.08 [0.007–0.96], p-value = 0.046). In the multivariate analysis for asymptomatic and microscopic recurrences, engaging in outdoor activities was found to be protective factors. Working as lumberjack, fisherman o farmer reduced the risk by 53% (95% CI [0.22–0.99], p-value = 0.048) for asymptomatic episodes and 35% (95% CI [0.47–0.91], p-value = 0.011) for microscopic episodes.

### Relative contribution of relapses to *P*. *vivax* recurrences

**Fig 5:**
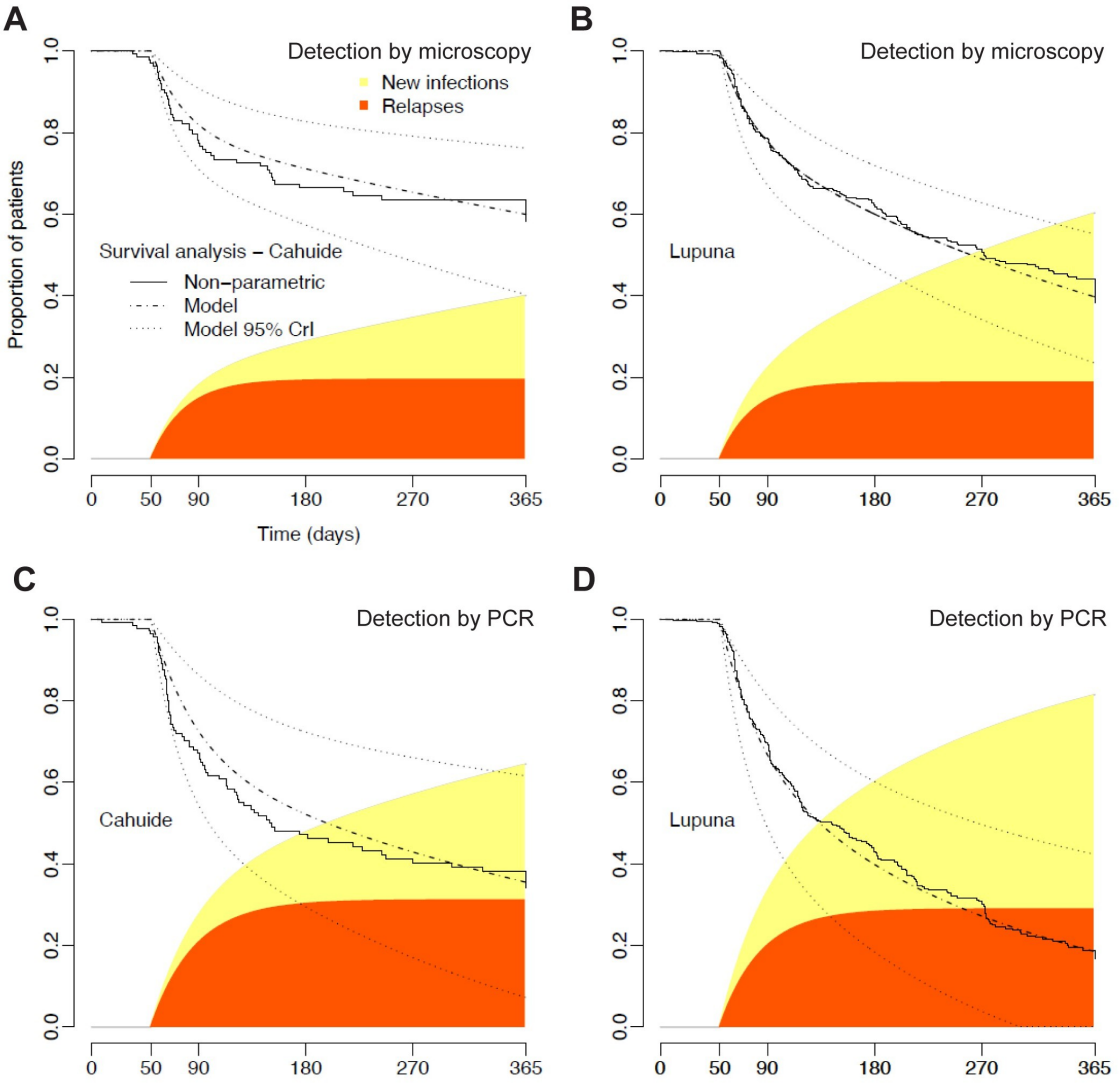
**Competing risk survival model applied to survival data from CAH and LUP.** Continuous lines represent the non-parametric Kaplan-Meier survival function for *P*. *vivax* recurrences detected by microscopy (A and B) and PCR (C and D), while the dashed lines represent the fitting of a competing risk survival model to empirical data. The black area of the curve represents the cumulative proportion of patients experiencing *P*. *vivax* relapses following treatment (fast dynamics) and the light grey area represents the cumulative proportion of patients experiencing new infections (slow dynamics).

We use a simple parametric modeling approach to capture the dynamics of *P*. *vivax* recurrences and to estimate the proportion of them that are due to relapses [27]. For the time to first recurrence detected by MIC, we estimate that 20% (95%CI 13–27%) of CAH and 19% (95%CI 12–27%) of LUP participants had one or more relapses during 12 months of follow-up. Therefore, relapses were estimated to account for 47% (95%CI 31–65%) and 31% (95%CI 20–43%) of the first microscopy-detected *P*. *vivax* recurrence in CAH and LUP, respectively (Fig 5A and 5B). We next focus on PCR-detected recurrences. We estimate that 31% (95%CI 20–45%) of the study participants in CAH and 29% (95%CI 18–42%) in LUP had one or more PCR-detected relapses over 12 months. We estimate that relapses account for 48% (95%CI 30–68%) and 35% (95%CI 22–51%) of the first PCR-detected *P*. *vivax* recurrence in CAH and LUP, respectively, observed over 12 months of follow-up (Fig 5C and 5D).

### Dynamics and evolution of *P*. *vivax* recurrence infections

This study describes eight scenarios of recurrence dynamics based on the incidence density (ID), which is the rate at which new cases of an outcome occur in a population over a specific period (Table 4). The first four scenarios focused on individuals who developed a single type of infection throughout the entire follow-up period. It was more common for individuals with recurrent microscopic infections to develop symptoms (ID = 53.7) than to experience symptomless episodes (ID = 2.6). Interestingly, individuals who did not receive treatment and only developed asymptomatic recurrences were moderately common (ID = 13.1). This suggests a significant role played by the immune response developed during previous malaria episodes before the cohort began. In contrast, a comparable number of individuals developed a mixed type of infections based on both diagnostic method (microscopic/submicroscopic) and clinical status (asymptomatic/symptomatic), with no clear pattern discernible (ID = 12.7). This highlights the heterogeneous response to recurrences among individuals and suggests a relatively short-term immune memory on this population.

**Table 4:** The scenarios describe the pattern of malaria infection over time at an individual level. This includes individuals with only one type of infection and those with an evolution of infection. Incidence density is described as new cases per 100 persons at risk.

Scenarios	N	% (Total = 385)	Incidence Density (incidence rate = 228.9)
**Scenarios with only one type of infections**			
Microscopic and Symptomatic infections	123	31.9	53.7
Microscopic and Asymptomatic infections	6	1.6	2.6
Individuals without treatment and only asymptomatic infections	30	7.8	13.1
Mixed mic/submic and asym/sym infections	29	7.5	12.7
**Evolution scenarios**			
Individuals with PCR+ become negative and remained negative	103	26.8	45
Individuals with symptomatic infections become asymptomatic infection	60	15.6	26.2
Individuals with microscopic infections become submicroscopic infection	32	8.3	14
Individuals with infections that evolved into submicroscopic and then resolved	22	5.7	9.6

**Fig 6:**
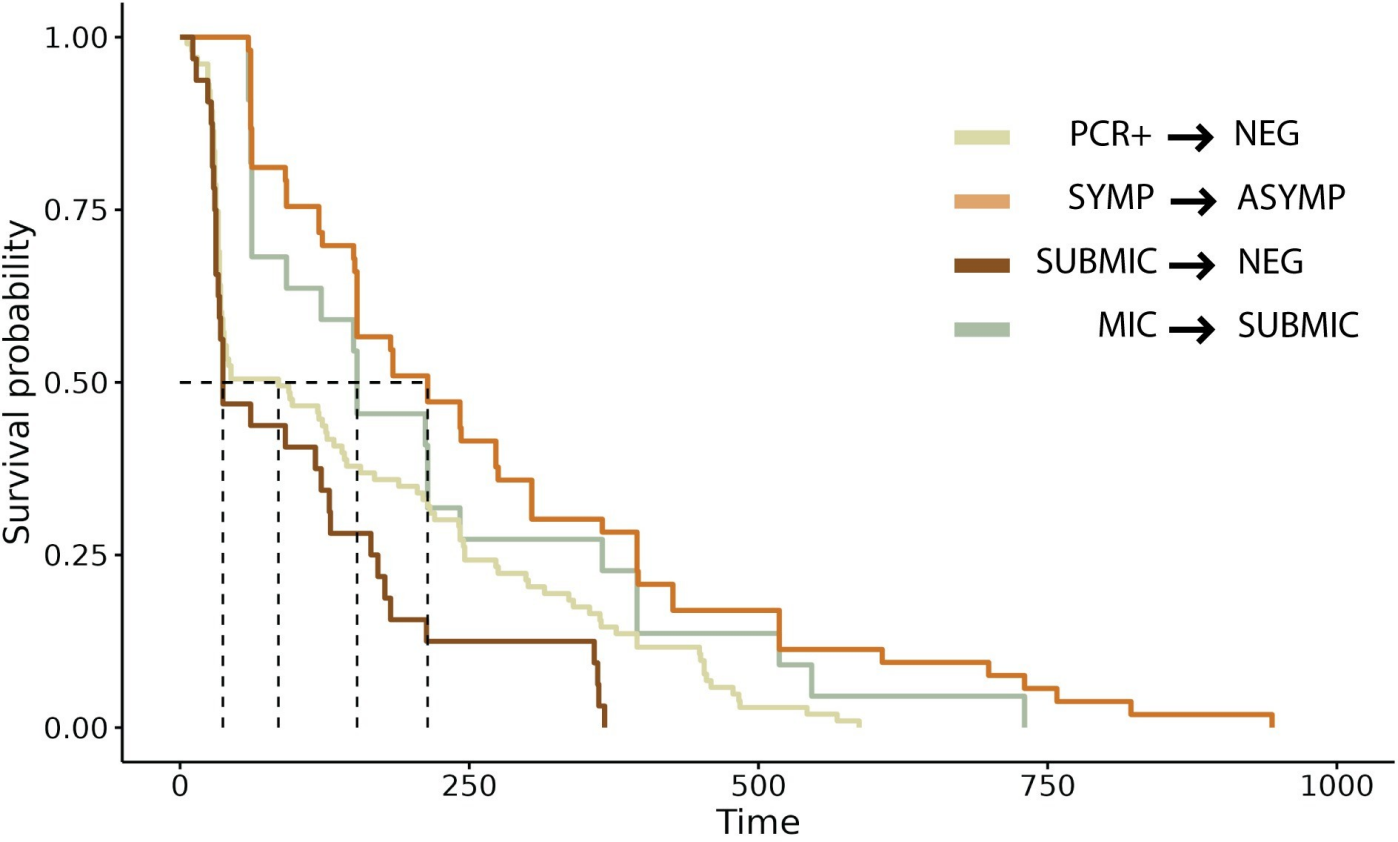
The evolution and resolution times of four epidemiological scenarios. This includes individuals with PCR + to PCR–or undetectable infection, symptomatic to asymptomatic infection, submicroscopic to undetectable infection, and microscopic to microscopic infection.

The second four scenarios focused on individuals with specific infections based on clinical status and diagnostics that evolve over time (Fig 1 and Table 4). In the first evolution scenario, 103 (26.8%) individuals were diagnosed as PCR-positive and became negative/undetectable over time. The median time to resolution of infection was 91 days (IQR [43.0, 236.0]) (Fig 6). For the second scenario, 60 individuals with only symptomatic infection progressed to asymptomatic during follow-ups. The ID for this scenario was 26.2 new cases per 100 person-years of follow-up, and the median time to evolution was estimated at 214 days (IQR [120.0, 395.0]) (Table 4 and Fig 6). In the third scenario, 32 individuals with microscopic infections evolved to submicroscopic infections. The ID for this scenario was 14 new cases per 100 person-years of follow-up, and the median time to evolution was 153 days (IQR [62.0, 334.25]) (Table 4 and Fig 6). In the last scenario, 22 individuals who started with submicroscopic recurrences evolved to be negative/indetectable over time. The ID for this scenario was 9.6 new cases per 100 person-years of follow-up and the median time to resolution was 45 days (Table 4 and Fig 6).

## Discussion

This study underscores the significant role that *Plasmodium vivax* recurrences play in the persistence and dynamics of the parasite within particular communities of the Peruvian Amazon. Characterized by low parasitemia, these recurrences are primarily detected only by PCR, with the majority being asymptomatic. In this region, malaria transmission shows high heterogeneity, varying with sociodemographic and geographic patterns among different communities [17, 23]. For instance, Lupuna community experienced intense seasonal transmission and constant floods between 2012 and 2013 [23]. During the 3-year follow-up period, a considerable number of individuals underwent multiple episodes of *P*. *vivax* infection, with short intervals between recurrences. Most of these recurrences were asymptomatic and submicroscopic, underscoring the ability of such infections to sustain transmission even in low-prevalence areas. Furthermore, individuals from Lupuna, exposed more frequently to the parasite, often had an extensive history of previous malaria infections, this likely facilitated immunity against local parasite strains [28]. Increased transmission rates were linked to marked seasonality in *P*. *vivax* gametocyte carriage, potentially explaining parasite transmission in this area [15].

Early diagnosis with a sensitive tool and treatment are pivotal in areas with residual malaria like the Peruvian Amazon, to halt transmission. This study reaffirms that molecular diagnosis using PCR is considerably more effective than microscopy in detecting recurrences [17, 25]. In both CAH and LUP, PCR identified a higher percentage of *P*. *vivax* infections, revealing over 74% of submicroscopic recurrences and 83% with asymptomatic recurrences. Traditional surveillance methods of the Peruvian Ministry of Health have been unable to detect these infections, which may significantly impact parasite transmission and mosquito infections [29–33]. Moreover, PCR allows for the earlier detection of recurrences, irrespective of their symptomatology, identifying the first recurrence within 5 months. Studies in South American countries with an epidemiological profile similar to that of Peru have reported similar detection times. In Brazil, a study involving nearly 30,000 potential recurrences found that the median time to the first recurrence was 71 days, and 69 days when all recurrences were included [34]. Similarly, in Colombia, a follow-up of nearly six months with 134 patients determined that the average time to the first recurrence ranged from 51 to 110 days [35]. On the other hand, in Myanmar, a one-year follow-up determined that the median time to the first recurrence was nearly 3 months, and this period decreased with subsequent recurrences [36]. Several factors contribute to the high recurrence rate detected by PCR including asymptomatic individuals and the rapid acquisition of clinical immunity to *P*. *vivax* infections [37]. When patients who are receiving treatment for malaria were experimentally infected with *P*. *vivax*, effective clinical immunity, even against heterologous challenge, was often attained as few as one to five infections [6]. Under natural exposure, clinical immunity to *P*. *vivax* is also acquired significantly more rapidly than to *P*. *falciparum* not only in high transmission settings but also in lower transmission settings such as Thailand [38]. The main driver of this rapid acquisition of immunity is the highest force of infections [39], caused largely by genetically distinct but related relapses [40] that account for up 80% of all *P*. *vivax* infections [41].

Recurrences of *P*. *vivax* have three origins [42]: (i) the activation of hypnozoites, the latent phase of the parasite in the liver, which generates relapses during the following weeks or even months after treatment; (ii) the recrudescence of the infection, which is the reappearance of blood stages following treatment, due to the incomplete parasite elimination; and (iii) reinfection by a new parasite after treatment. However, despite the advances in sequencing and bioinformatics tools, determining the origin of the recurrence is still complicated. In the case of the Amazon, recurrence rates ranged from 10.3% to 57%, considering that many patients experienced more than one episode during follow-ups [43–47]. One particular concern is relapses since their activation mechanism is still unknown. However, it has been suggested that systemic infections by parasites or bacteria can activate hypnozoites [42, 48]. Also, it has been determined that large inoculations of sporozoites and the absence of treatment increase the probability of relapses [49]. Additionally, there have been described homologous relapses (where the genotype of the first infection is the same as the genotype of the recurrence) and heterologous relapses (where the genotype of the first infection is different from the genotype of the recurrence). In this study, we aimed to use mathematical modeling to unveil the significant role of relapses in maintaining transmission, even in areas with low transmission where submicroscopic and asymptomatic infections prevail. We found that relapses contribute to 48% of the first PCR-detected *P*. *vivax* recurrence in CAH and 35% in LUP over a 12-month follow-up period. These proportions are notably high compared to other studies in the Amazon region. For instance, one study followed 302 patients infected with *P*. *vivax* in 25 communities in Loreto for 2 years to determine the origin of their recurrence [47]. Within the first 28 days of follow-up, no recrudescences were observed, but the reinfection rate was 3.7%. After day 28, 4% of homologous recurrences were relapses and 5% were reinfections, while among heterologous recurrences, 11.3% were relapses and 3.6% were reinfections. In Brazil, it has been estimated that pregnant women have a higher risk of relapses compared to control individuals treated with primaquine [27]. The relapse rates can range from 11.4% in the control group to 28.3% in pregnant women, causing their first malaria recurrence within 12 months after the baseline episode [27].

An important part of the analysis involved determining which sociodemographic factors are associated with the detection time of recurrences. It was found that the community of origin, age, the number of previous malaria infections, and occupation were associated with recurrences. Initially, significant differences were observed in the sociodemographic characteristics of both communities, primarily in resource availability. The effect of poor housing conditions on malaria incidence is well documented, as low-income individuals cannot afford effective preventive measures [22, 50]. Additionally, in Lupuna, the main activity is agriculture, while Cahuide has been established as a deforestation area, predisposing it to the formation of mosquito breeding sites [22]. In both scenarios, there is a risk of parasite exposure and, consequently, recurrences. Interestingly, we found that individuals in Lupuna have a higher probability of experiencing symptomatic and microscopic infections, while the likelihood of submicroscopic infections is reduced. Another factor associated with submicroscopic recurrences is age group, with individuals older than 15 years showing a lower probability of developing submicroscopic recurrences. Parasitemia has been shown to be age-dependent, declining over time. Under conditions of chronic exposure, it is suggested that adults develop antiparasitic and clinical immunity more rapidly than children or adolescents [6, 51]. However, in conditions of acute exposure, such as in the Peruvian Amazon, adults present a higher risk of contracting malaria [6, 51]. Thus, the age at which an individual is first exposed to malaria is an important factor in the natural acquisition of malaria immunity [6]. We also found an association between previous episodes of *P*. *vivax* and submicroscopic recurrences. This factor is likely related to the development of immunity in the population, a consequence of high exposure to and infection with *P*. *vivax* [52]. This aligns with the epidemiological context during the data collection period, which coincided with a malaria peak in the Peruvian Amazon [9]. For instance, a study conducted by Pham et al. in Vietnam concluded that the main risk factor for microscopic and symptomatic recurrences was previous infection with *P*. *falciparum*, a factor not evaluated in this study despite the presence of positive cases of *P*. *falciparum*, as both species coexist in the region [53]. Other studies on risk factors for recurrence have found different results, which can be explained by the unique epidemiological context of each country. In Brazil, the risk of recurrence was found to decrease with increasing age [34]. Factors associated with shorter time to recurrence included individuals younger than 3 years, male sex, absence of hypnozoite treatment, and domestic occupation [34]. Conversely, in Thailand [36] and Colombia [35], men had a higher risk of experiencing multiple episodes of malaria compared to women; however, this was not observed in our study.

Finally, the study’s detailed analysis of parasite dynamics and its relationship with clinical outcomes sheds light on individual immune responses and regional malaria transmission patterns. This study evaluated different infection scenarios focusing on individuals with only one type of infection and individuals with evolution scenarios. The most common scenario includes individuals who experienced only symptomatic and microscopic infections, reflecting the peak of cases in the region during those years [23]. This observation also suggests a rapid loss of immunity, likely due to a decline in antibody titers against asexual stages and impairment of the acquired immune system [54]. The second most common scenario was observed in individuals with positive infections that eventually resolved. For instance, a study conducted in Vietnam, focusing on the persistence of submicroscopic *P*. *vivax* infection, revealed that the median duration of infection was 6 months and individuals had a 59% chance of having parasitemia for 4 months or longer [55]. The oscillation and persistence of parasitemia can continue for weeks, even when individuals do not exhibit clinical symptoms and do not seek treatment [16, 29, 53]. Interestingly, in our study, the duration of infection was 3 months, which differs from what was previously described, likely due to different epidemiological conditions. The undetected parasitemia state, which is associated with low density parasitemia, is generally characterized by a mild or even asymptomatic infection [56, 57]. In adults, the rate of recovery is influenced by several factors besides a strong humoral immune response, risky behaviors, and the presence of other systemic diseases [58]. Another common scenario in our cohort involves individuals who initially had symptomatic infections that later changed to asymptomatic. It is intriguing to observe how these individuals undergo changes in their clinical responses during their recurrences. While it is more probable that these asymptomatic episodes are due to reinfection, estimating this probability is challenging, particularly for multiple recurrences. The role of these asymptomatic infections, with detected parasitemia by PCR, in maintaining *P*. *vivax* transmission is not yet well understood and, in some studies, carried out in Peru and Brazil have shown different results. Most, if not all, of these asymptomatic infections produce gametocytes [15, 32]. Nevertheless, the asymptomatic cases are infectious to anopheline vectors, although at a substantially lower level than clinical cases [29, 30] and may contribute substantially to maintaining *P*. *vivax* transmission given the high prevalence of the “silent” infections. When analyzing the disease evolution pattern, it is important to consider the population genetics of the surrounding parasites. The genetic diversity of these parasites not only influences regional genetic flow but also contributes significantly to intra-host variability, which is particularly complex in the case of *P*. *vivax* infection [59].

The primary strength of this study lies in its prospective cohort design, characterized by rigorous follow-up procedures, and the integration of epidemiological, parasitological, and modeling data. However, it is important to acknowledge certain limitations inherent to the fixed cohort design. Since individuals experience initial *P*. *vivax* infections at different points in time, the duration of follow-up varies from one person to another, potentially influencing the accuracy of recurrence estimations. Nonetheless, the inclusion criteria elucidated in the methods section contribute to the generation of robust results.

## Conclusion

In conclusion, the *P*. *vivax* recurrences observed in CAH and LUP over the 3-year period are multifaceted, influenced by distinct intrinsic community factors. Molecular diagnostics has shown that asymptomatic parasitic reservoirs are more widespread than previously thought and have emerged as a prevalent and critical scenario for transmission. Detectability of asymptomatic malaria infections and the relevance of submicroscopic infections for parasite transmission to mosquitoes and for community interventions that aim at reducing transmission is also important. Deployment of molecular diagnostic tools is needed to provide adequate insight into the epidemiology of malaria and infection dynamics to aid elimination efforts. These findings underscore the challenges of eradicating the disease from the Peruvian Amazon and emphasize the importance of comprehensive monitoring and control strategies.

## Supporting information

S1 Fig Scheme of the individual selection model whereby, following one or multiple PCR+, they achieve the resolution of the infection. S1 Fig shows the selection process of individuals with only PCR- events after a period of PCR+. First, the time in which the individuals had only PCR- events were analyzed and the times greater than the 0.75 quantiles plus two times the interquartile range and that had a period of symptomatic episodes at the beginning were selected. Finally, the time of these asymptomatic episodes was plotted before resolving the infection with the Kaplan-Meier curve.
(TIFF)

S2 Fig Scheme of the individual selection model whereby, following one or multiple symptomatic events, and they change to asymptomatic. S2 Fig shows the selection process of individuals with only asymptomatic events after a period of symptomatic infections. First, the time in which the individuals had only asymptomatic events were analyzed and the times greater than the 0.75 quantiles plus two times the interquartile range and that had a period of symptomatic episodes at the beginning were selected. Finally, the time of these asymptomatic episodes was plotted before resolving the infection with the Kaplan-Meier curve.
(TIFF)

S3 Fig Scheme of the individual selection model whereby, following one or multiple microscopic events, they change to submicroscopic events. S3 Fig shows the selection process of individuals with only sub microscopic events after a period of symptomatic infections. First, the time in which the individuals had only microscopic events were analyzed and the times greater than the 0.75 quantiles plus two times the interquartile range and that had a period of symptomatic episodes at the beginning were selected. Finally, the time of these asymptomatic episodes was plotted before resolving the infection with the Kaplan-Meier curve.
(TIFF)

S4 Fig Scheme of the individual selection model whereby, following one or multiple submicroscopic events, they achieve the resolution of the infection. S4 Fig shows the selection process of individuals who manage to resolve the infection after a period of submicroscopic infections. First, the time in which the individuals had only PCR- events were analyzed and the times greater than the 0.75 quantiles plus two times the interquartile range and that had a period of submicroscopic episodes at the beginning were selected. Finally, the time of these submicroscopic episodes was plotted before resolving the infection with the Kaplan-Meier curve.
(TIFF)

S5 Fig Diagram of symptoms present in *P*. *vivax* infected individuals according to clinical status (asym /sym) and diagnostic status (mic/submic). (TIF)

S6 Fig The cumulative risk of developing recurrent *Plasmodium vivax* infections by PCR and microscopy detection, according to community, sex, age group, outdoor occupation, and previous malaria episodes. Comparison between survival curves was measured using the Log-Rank test.
(TIF)

S7 Fig The cumulative risk of developing recurrent *Plasmodium vivax* infections by clinical status (asymptomatic or symptomatic) according to community, sex, age group, outdoor occupation, and previous malaria episodes. Comparison between survival curves was measured using the Log-Rank test.
(TIF)

S1 Table Characterization of symptoms present in recurrences Asym/Sym and Sub/Mic. (DOCX)

S2 Table Univariate analyses of risk factors according to clinical status (asym/sym) and diagnostic status (mic/submic). (DOCX)

S3 Table Adjusted risk factor analysis according to clinical status (asym/sym) and diagnostic status (mic/submic). Multivariate logistic regression analysis of Cox Counting Process for multiple recurrences. HR values (hazard ratios) less than 1 represent protective factors and values greater than 1 represent risk factors.
(DOCX)

S1 Data Raw dataset of the cohort used for analysis. (XLSX)
