# Supplementary figures and images for: Recurrence patterns and evolution of submicroscopic and asymptomatic Plasmodium vivax infections in malaria-endemic areas of the Peruvian Amazon
Source: PLoS Negl Trop Dis. 2024 Oct 31;18(10):e0012566. doi: 10.1371/journal.pntd.0012566 (PMC11527163; doi:10.1371/journal.pntd.0012566)

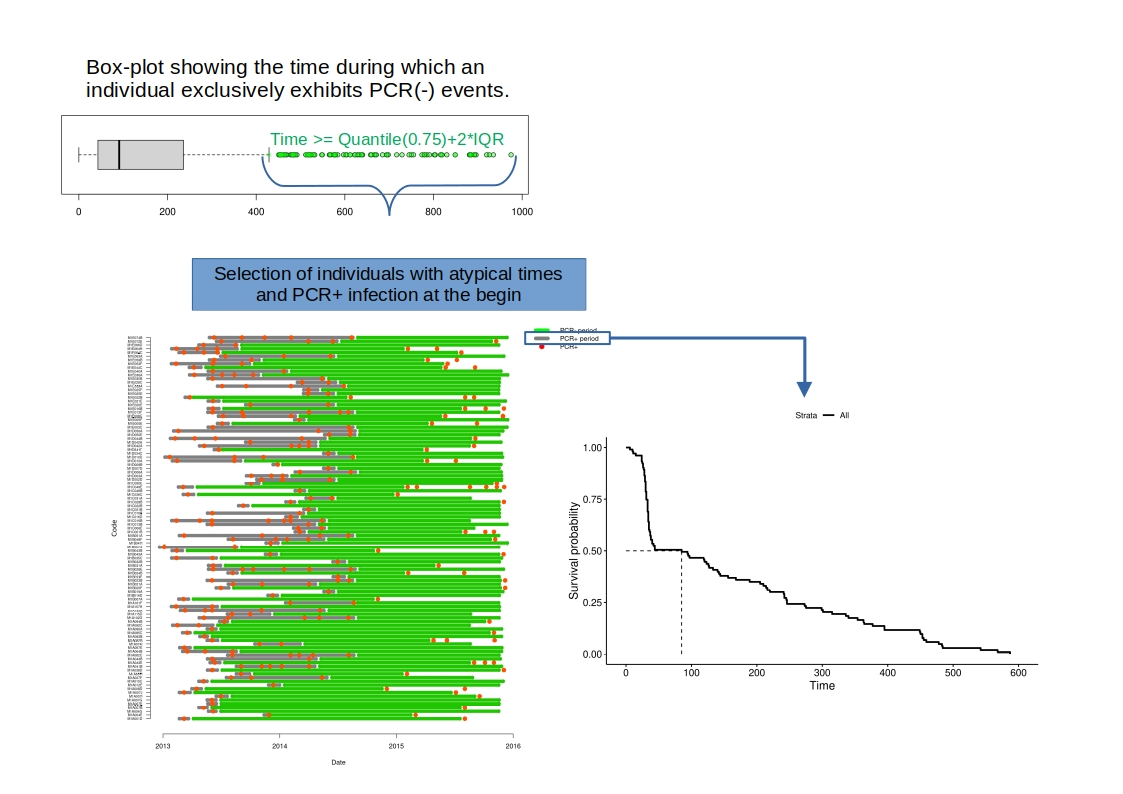

Supplement: S1 Fig — shows the selection process of individuals with only PCR- events after a period of PCR+. First, the time in which the individuals had only PCR- events were analyzed and the times greater than the 0.75 quantiles plus two times the interquartile range and that had a period of symptomatic episodes at the beginning were selected. Finally, the time of these asymptomatic episodes was plotted before resolving the infection with the Kaplan-Meier curve. (TIFF) [file pntd.0012566.s001.tiff]

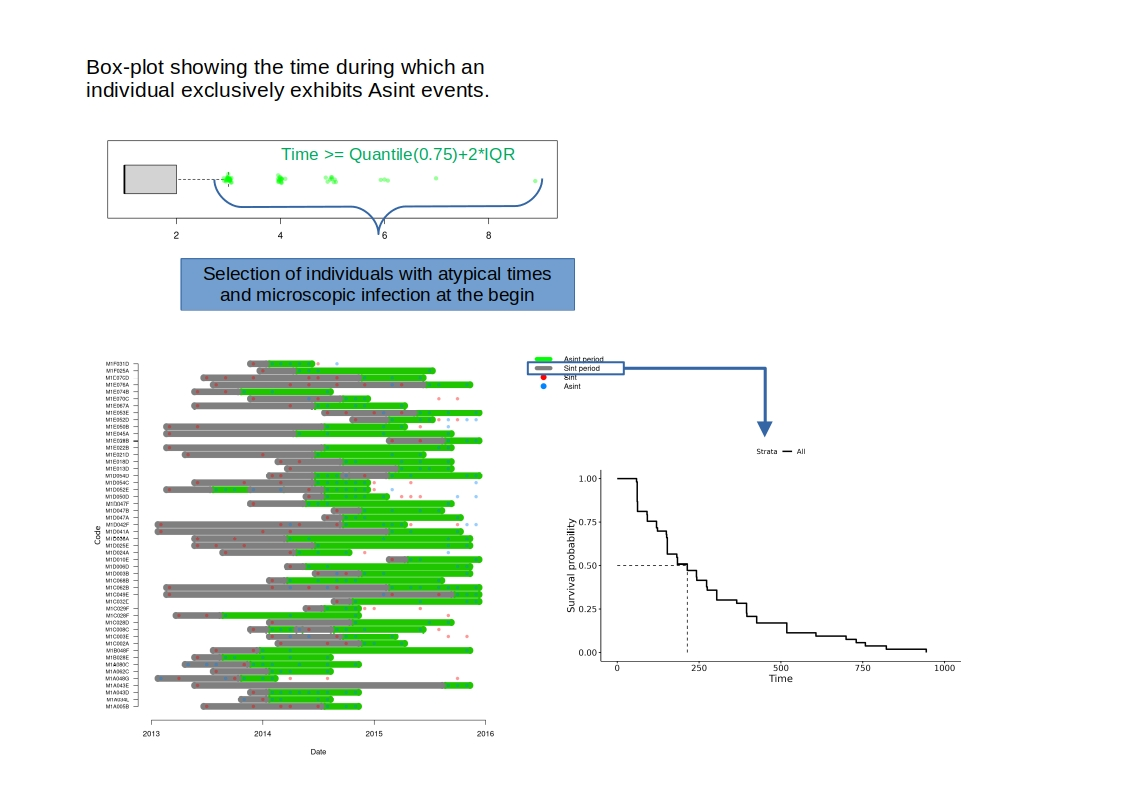

Supplement: S2 Fig — shows the selection process of individuals with only asymptomatic events after a period of symptomatic infections. First, the time in which the individuals had only asymptomatic events were analyzed and the times greater than the 0.75 quantiles plus two times the interquartile range and that had a period of symptomatic episodes at the beginning were selected. Finally, the time of these asymptomatic episodes was plotted before resolving the infection with the Kaplan-Meier curve. (TIFF) [file pntd.0012566.s002.tiff]

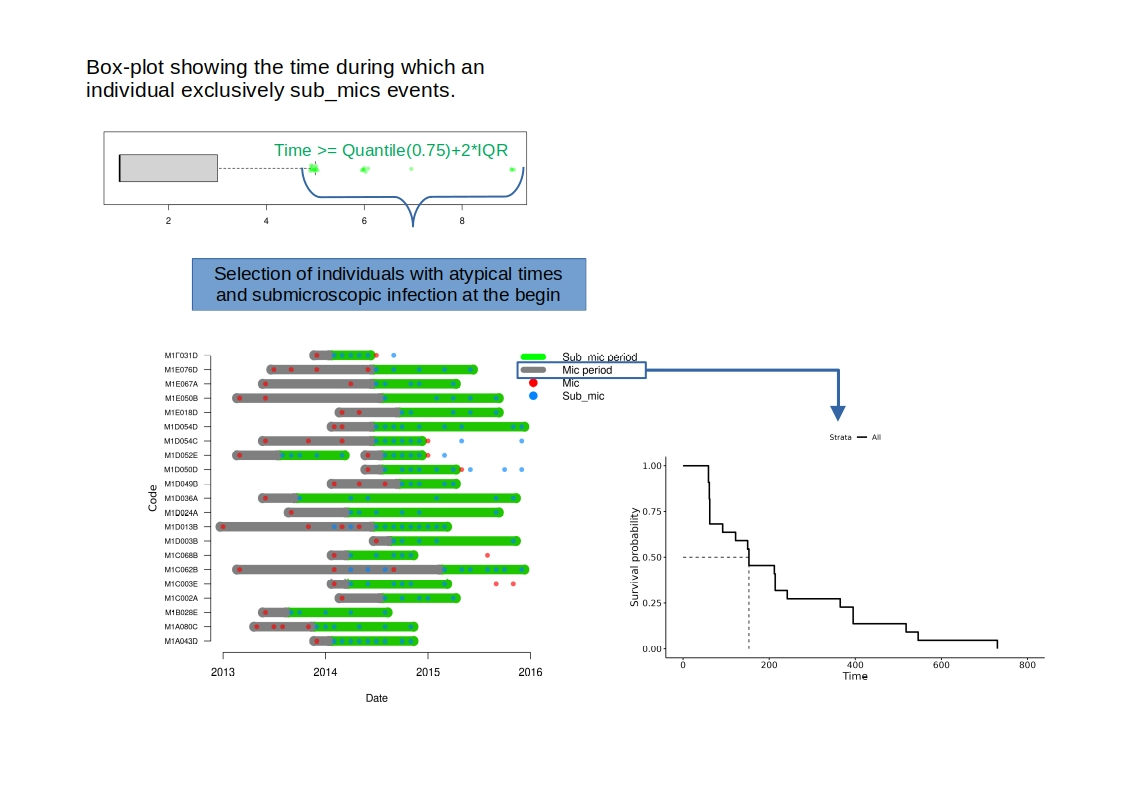

Supplement: S3 Fig — shows the selection process of individuals with only sub microscopic events after a period of symptomatic infections. First, the time in which the individuals had only microscopic events were analyzed and the times greater than the 0.75 quantiles plus two times the interquartile range and that had a period of symptomatic episodes at the beginning were selected. Finally, the time of these asymptomatic episodes was plotted before resolving the infection with the Kaplan-Meier curve. (TIFF) [file pntd.0012566.s003.tiff]

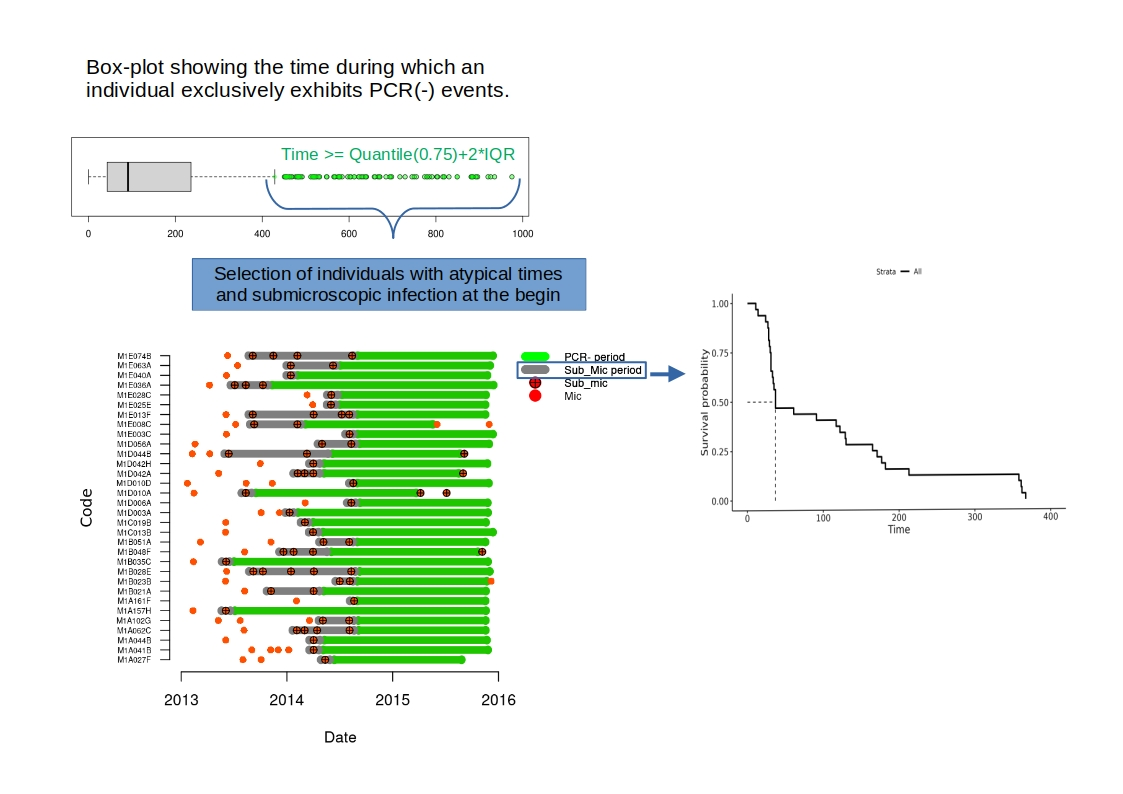

Supplement: S4 Fig — shows the selection process of individuals who manage to resolve the infection after a period of submicroscopic infections. First, the time in which the individuals had only PCR- events were analyzed and the times greater than the 0.75 quantiles plus two times the interquartile range and that had a period of submicroscopic episodes at the beginning were selected. Finally, the time of these submicroscopic episodes was plotted before resolving the infection with the Kaplan-Meier curve. (TIFF) [file pntd.0012566.s004.tiff]

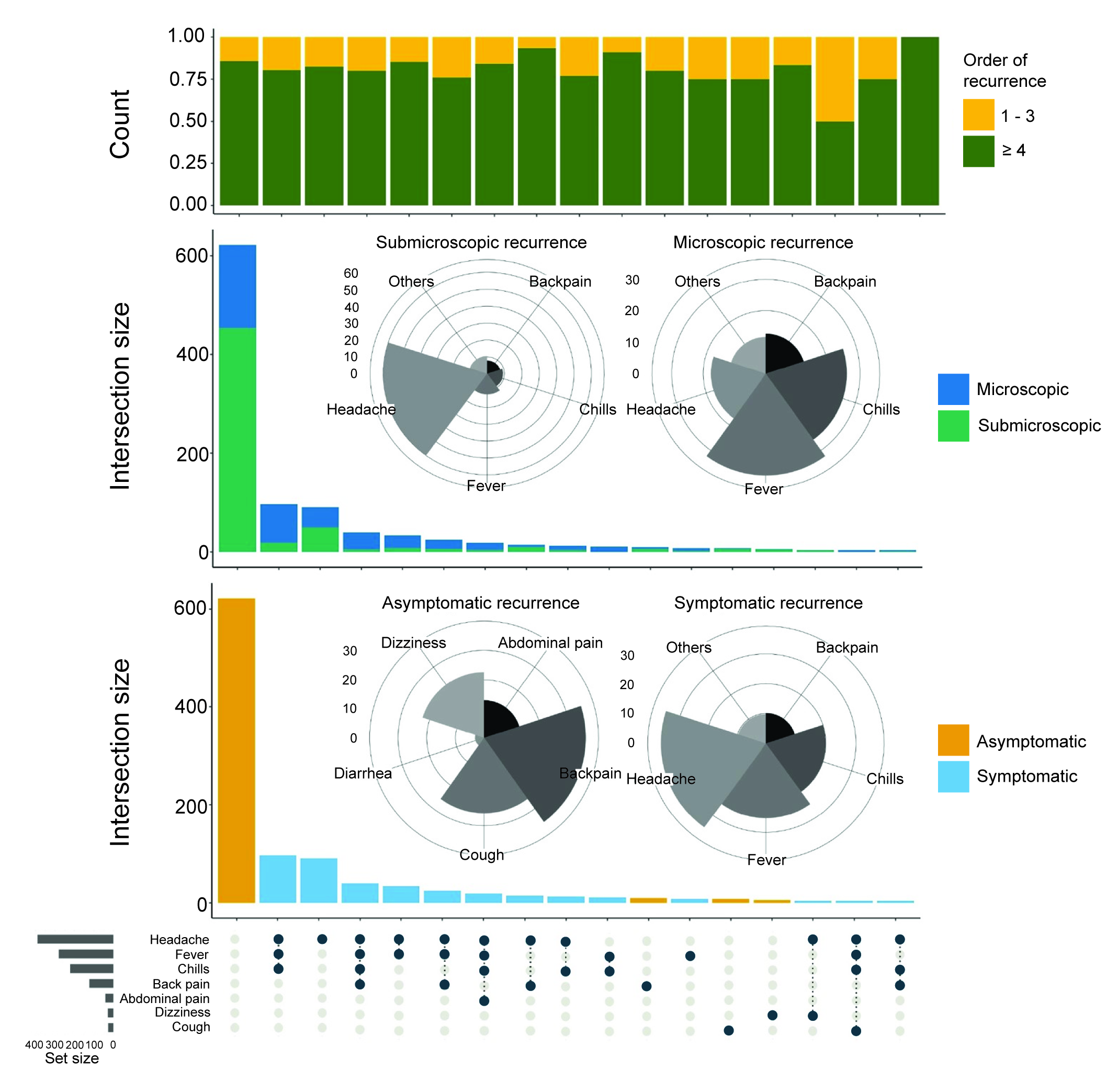

Supplement: S5 Fig — (TIF) [file pntd.0012566.s005.tif]

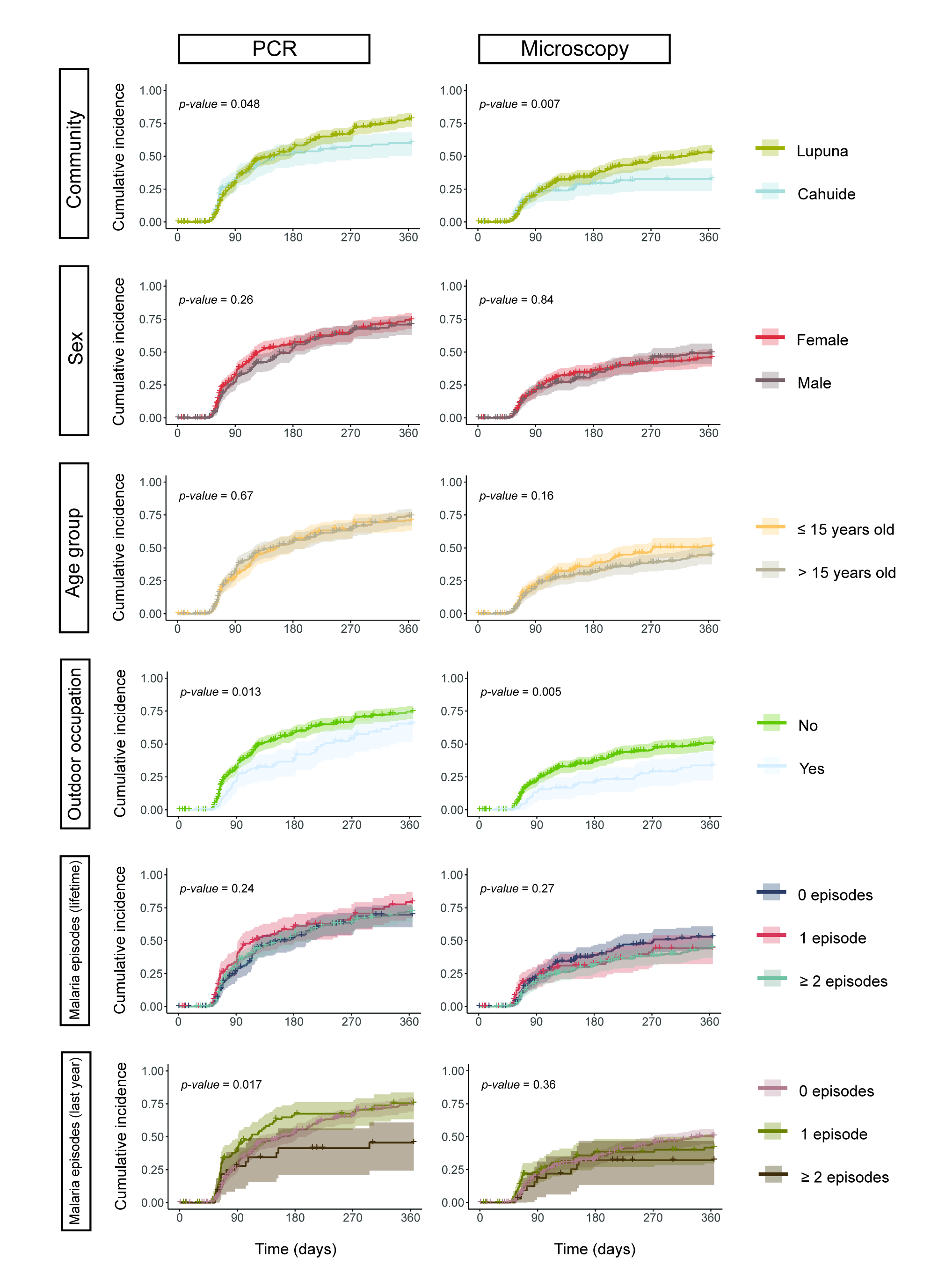

Supplement: S6 Fig — Comparison between survival curves was measured using the Log-Rank test. (TIF) [file pntd.0012566.s006.tif]

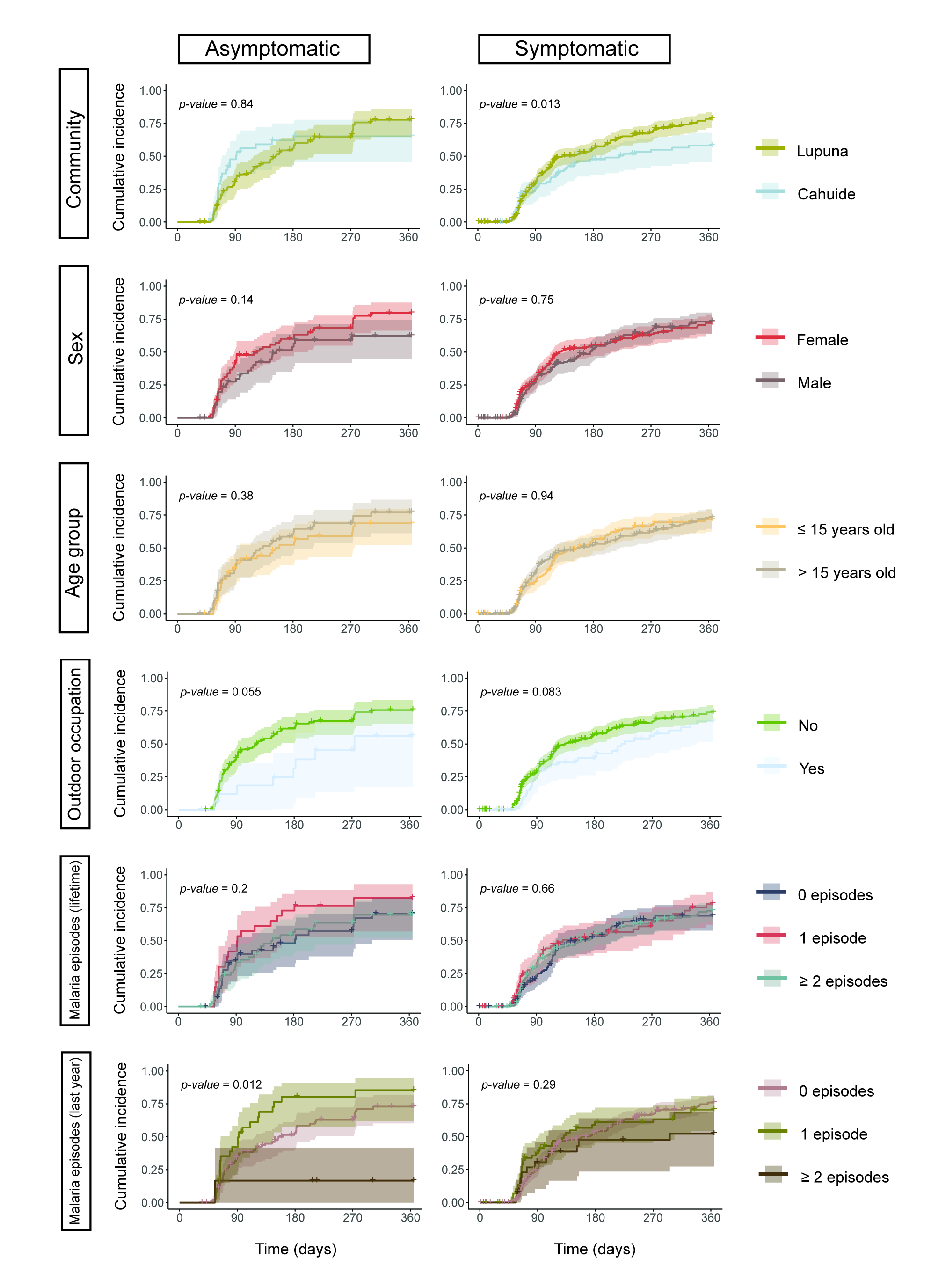

Supplement: S7 Fig — Comparison between survival curves was measured using the Log-Rank test. (TIF) [file pntd.0012566.s007.tif]
