# Supplementary material for: Recurrence patterns and evolution of submicroscopic and asymptomatic Plasmodium vivax infections in malaria-endemic areas of the Peruvian Amazon
Source: PLoS Negl Trop Dis. 2024 Oct 31;18(10):e0012566. doi: 10.1371/journal.pntd.0012566 (PMC11527163; doi:10.1371/journal.pntd.0012566)
Supplement: S1 Table — (DOCX) [file pntd.0012566.s008.docx]

**S1 Table.** **Characterization of symptoms present in recurrences Asym/Sym and Sub/Mic.**

|  | **Asymptomatic** | | **Symptomatic** | | **Submicroscopic** | | **Microscopic** | |
| --- | --- | --- | --- | --- | --- | --- | --- | --- |
| **Symptom** | **n = 651** | **%** | **n = 417** | **%** | **n = 601** | **%** | **n = 467** | **%** |
| Fever | 0 | 0.00 | 281 | 67.39 | 55 | 9.15 | 226 | 48.39 |
| Chills | 0 | 0.00 | 222 | 53.24 | 41 | 6.82 | 181 | 387.58 |
| Headache | 0 | 0.00 | 390 | 93.53 | 123 | 20.47 | 267 | 57.17 |
| Sickness | 0 | 0.00 | 8 | 1.92 | 0 | 0.00 | 8 | 1.71 |
| Vomits | 0 | 0.00 | 9 | 2.16 | 0 | 0.00 | 9 | 1.93 |
| Anorexia | 0 | 0.00 | 6 | 1.44 | 2 | 0.33 | 4 | 0.86 |
| Dizziness | 7 | 1.08 | 22 | 5.28 | 16 | 2.66 | 13 | 2.78 |
| Diarrhea | 1 | 0.15 | 6 | 1.44 | 3 | 0.50 | 4 | 0.86 |
| Cough | 8 | 1.23 | 19 | 4.56 | 11 | 1.83 | 16 | 3.43 |
| Abdominal pain | 4 | 0.61 | 37 | 8.87 | 15 | 2.50 | 26 | 5.57 |
| Back pain | 11 | 1.69 | 113 | 27.10 | 35 | 5.82 | 89 | 19.06 |
| Rash | 0 | 0.00 | 2 | 0.48 | 0 | 0.00 | 2 | 0.43 |
| Other | 0 | 0.00 | 0 | 0.00 | 0 | 0.00 | 0 | 0.00 |
