# Supplementary material for: Recurrence patterns and evolution of submicroscopic and asymptomatic Plasmodium vivax infections in malaria-endemic areas of the Peruvian Amazon
Source: PLoS Negl Trop Dis. 2024 Oct 31;18(10):e0012566. doi: 10.1371/journal.pntd.0012566 (PMC11527163; doi:10.1371/journal.pntd.0012566)
Supplement: S2 Table — (DOCX) [file pntd.0012566.s009.docx]

**S2 Table.** **Univariate analyses of risk factors according to** **clinical status (asym/sym) and diagnostic status (mic/submic).**

| **Factor** | **Asymptomatic** | | | **Symptomatic** | | | **Submicroscopic** | | | **Microscopic** | | |
| --- | --- | --- | --- | --- | --- | --- | --- | --- | --- | --- | --- | --- |
|  | **HR** | **95% IC** | ***p-value*** | **HR** | **95% IC** | ***p-value*** | **HR** | **95% IC** | ***p-value*** | **HR** | **95% IC** | ***p-value*** |
| **Community** | | | | | | | | | | | | |
| Cahuide | 1 |  |  | 1 |  |  | 1 |  |  | 1 |  |  |
| Lupuna | 1.1 | 0.66 - 1.7 | 0.821 | 1.6 | 1.1 – 2.1 | 0.006 | 0.69 | 0.22 – 2.2 | 0.522 | 1.4 | 1.1 – 1.9 | 0.009 |
| **Sex** | | | | | | | | | | | | |
| Female | 1 |  |  | 1 |  |  | 1 |  |  | 1 |  |  |
| Male | 0.72 | 0.46 - 1.1 | 0.154 | 0.99 | 0.77 - 1.3 | 0.97 | 1.7 | 0.51 – 5.5 | 0.403 | 0.89 | 0.71 - 1.1 | 0.31 |
| **Age groups** | | | | | | | | | | | | |
| ≤ 15 years | 1 |  |  | 1 |  |  | 1 |  |  | 1 |  |  |
| > 15 years | 1.3 | 0.84 - 2 | 0.243 | 1 | 0.79 - 1.3 | 0.836 | 0.29 | 0.091 – 0.89 | 0.031 | 1.1 | 0.91 - 1.4 | 0.235 |
| **Outdoor occupation (lumberjack, fisherman o farmer)** | | | | | | | | | | | | |
| No | 1 |  |  | 1 |  |  | 1 |  |  | 1 |  |  |
| Yes | 0.82 | 0.59 – 1.1 | 0.241 | 0.79 | 0.53 - 1.2 | 0.243 | 0.69 | 0.15 – 3.2 | 0.633 | 0.77 | 0.57 - 1 | 0.081 |
| **Malaria episodes by *Plasmodium vivax* in your lifetime** | | | | | | | | | | | | |
| 0 | 1 |  |  | 1 |  |  | 1 |  |  | 1 |  |  |
| 1 | 1.45 | 0.83 – 2.6 | 0.194 | 1.1 | 0.79 - 1.7 | 0.467 | 0.3 | 0.032 – 2.82 | 0.294 | 1.3 | 0.94 - 1.8 | 0.111 |
| ≥ 2 | 0.99 | 0.6 – 1.6 | 0.975 | 1.1 | 0.8 - 1.4 | 0.627 | 0.3 | 0.091 – 0.97 | 0.044 | 1.1 | 0.85 – 1.4 | 0.474 |
| **Malaria episodes by *Plasmodium vivax* in the last year** | | | | | | | | | | | | |
| 0 | 1 |  |  | 1 |  |  | 1 |  |  | 1 |  |  |
| 1 | 1.5 | 0.926 – 2.4 | 0.099 | 0.99 | 0.7 – 1.4 | 0.946 | 1.01 | 0.114 – 8.9 | 0.992 | 1.16 | 0.88 – 1.54 | 0.298 |
| ≥ 2 | 0.18 | 0.025 – 1.3 | 0.087 | 0.6 | 0.34 – 1.1 | 0.077 | 0.21 | 0.026 – 1.6 | 0.131 | 0.55 | 0.32 – 0.97 | 0.038 |
