# Supplementary material for: Recurrence patterns and evolution of submicroscopic and asymptomatic Plasmodium vivax infections in malaria-endemic areas of the Peruvian Amazon
Source: PLoS Negl Trop Dis. 2024 Oct 31;18(10):e0012566. doi: 10.1371/journal.pntd.0012566 (PMC11527163; doi:10.1371/journal.pntd.0012566)
Supplement: S3 Table — Multivariate logistic regression analysis of Cox Counting Process for multiple recurrences. HR values (hazard ratios) less than 1 represent protective factors and values greater than 1 represent risk factors. (DOCX) [file pntd.0012566.s010.docx]

**S3 Table. Adjusted risk factor analysis according to clinical status (asym/sym) and diagnostic status (mic/submic).** Multivariate logistic regression analysis of Cox Counting Process for multiple recurrences. HR values (hazard ratios) less than 1 represent protective factors and values greater than 1 represent risk factors.

| **Factor** | **Asymptomatic** | | | **Symptomatic** | | | **Submicroscopic** | | | **Microscopic** | | |
| --- | --- | --- | --- | --- | --- | --- | --- | --- | --- | --- | --- | --- |
|  | **HR** | **95% IC** | ***p-value*** | **HR** | **95% IC** | ***p-value*** | **HR** | **95% IC** | ***p-value*** | **HR** | **95% IC** | ***p-value*** |
| **Community** | | | | | | | | | | | | |
| Cahuide | 1 |  |  | 1 |  |  | 1 |  |  | 1 |  |  |
| Lupuna | 1.14 | 0.65 - 2.04 | 0.637 | 1.54 | 1.06 - 2.23 | 0.024 | 0.08 | 0.007 - 0.96 | 0.046 | 1.50 | 1.09 - 2.05 | 0.011 |
| **Age groups** | | | | | | | | | | | | |
| ≤ 15 years | 1 |  |  | 1 |  |  | 1 |  |  | 1 |  |  |
| > 15 years | 1.61 | 0.95 - 2.73 | 0.076 | 1.17 | 0.83 – 1.64 | 0.363 | 0.55 | 0.06 – 4.98 | 0.597 | 1.31 | 0.99 – 1.75 | 0.0596 |
| **Outdoor occupation (lumberjack, fisherman o farmer)** | | | | | | | | | | | | |
| No | 1 |  |  | 1 |  |  | 1 |  |  | 1 |  |  |
| Yes | 0.47 | 0.22 - 0.99 | 0.048 | 0.72 | 0.5 - 1.0 | 0.083 | 1.65 | 0.19 – 1.8 | 0.646 | 0.65 | 0.47 - 0.91 | 0.011 |
| **Malaria episodes by *Plasmodium vivax* in your lifetime** | | | | | | | | | | | | |
| 0 | 1 |  |  | 1 |  |  | 1 |  |  | 1 |  |  |
| 1 | 1.1 | 0.52 - 2.22 | 0.852 | 1.05 | 0.68 - 1.7 | 0.827 | 0.42 | 0.01 – 1.5 | 0. 636 | 1.10 | 0.76 - 1.60 | 0.601 |
| ≥ 2 | 0.88 | 0.48 – 1.64 | 0.696 | 1.1 | 0.75 – 1.63 | 0.625 | 1.63 | 0.16 – 1.7 | 0.680 | 1.03 | 0.74 - 1.44 | 0.841 |
| **Malaria episodes by *Plasmodium vivax* in the last year** | | | | | | | | | | | | |
| 0 | 1 |  |  | 1 |  |  | 1 |  |  | 1 |  |  |
| 1 | 1.7 | 0.89 - 3.22 | 0.106 | 1.12 | 0.72 - 1.73 | 0.625 | 1.95 | 0.08 – 4.9 | 0.685 | 1.28 | 0.89 - 1.84 | 0.167 |
| ≥ 2 | 0.22 | 0.03 – 1.66 | 0.142 | 0.72 | 0.38 – 1.35 | 0.302 | 0.04 | 0.001 – 0.96 | 0.047 | .0.65 | 0.35 – 1.18 | 0.160 |
